# Individual flowering phenology shapes plant–pollinator interactions across ecological scales affecting plant reproduction

**DOI:** 10.1002/ece3.9707

**Published:** 2023-01-04

**Authors:** Audrey Labonté, Lucie S. Monticelli, Mélinda Turpin, Emeline Felten, Emilien Laurent, Annick Matejicek, Luc Biju‐Duval, Chantal Ducourtieux, Eric Vieren, Violaine Deytieux, Stéphane Cordeau, David Bohan, Adam J. Vanbergen

**Affiliations:** ^1^ Agroécologie, INRAE, Institut Agro Univ. Bourgogne, Univ. Bourgogne Franche‐Comté Dijon France; ^2^ Université Côte d'Azur, INRAE, CNRS, UMR ISA Nice France; ^3^ Unité Expérimentale du Domaine d'Epoisses U2E, INRAE Bretenière France

**Keywords:** agroecological infrastructure, *Centaurea*, network structure, plant–insect community, seed set, spatio‐temporal heterogeneity

## Abstract

The balance of pollination competition and facilitation among co‐flowering plants and abiotic resource availability can modify plant species and individual reproduction. Floral resource succession and spatial heterogeneity modulate plant–pollinator interactions across ecological scales (individual plant, local assemblage, and interaction network of agroecological infrastructure across the farm). Intraspecific variation in flowering phenology can modulate the precise level of spatio‐temporal heterogeneity in floral resources, pollen donor density, and pollinator interactions that a plant individual is exposed to, thereby affecting reproduction. We tested how abiotic resources and multi‐scale plant–pollinator interactions affected individual plant seed set modulated by intraspecific variation in flowering phenology and spatio‐temporal floral heterogeneity arising from agroecological infrastructure. We transplanted two focal insect‐pollinated plant species (*Cyanus segetum* and *Centaurea jacea*, *n* = 288) into agroecological infrastructure (10 sown wildflower and six legume–grass strips) across a farm‐scale experiment (125 ha). We applied an individual‐based phenologically explicit approach to match precisely the flowering period of plant individuals to the concomitant level of spatio‐temporal heterogeneity in plant–pollinator interactions, potential pollen donors, floral resources, and abiotic conditions (temperature, water, and nitrogen). Individual plant attractiveness, assemblage floral density, and conspecific pollen donor density (*C. jacea*) improved seed set. Network linkage density increased focal species seed set and modified the effect of local assemblage richness and abundance on *C. segetum*. Mutual dependence on pollinators in networks increased *C. segetum* seed set, while *C. jacea* seed set was greatest where both specialization on pollinators and mutual dependence was high. Abiotic conditions were of little or no importance to seed set. Intra‐ and interspecific plant–pollinator interactions respond to spatio‐temporal heterogeneity arising from agroecological management affecting wild plant species reproduction. The interplay of pollinator interactions within and between ecological scales affecting seed set implies a co‐occurrence of pollinator‐mediated facilitative and competitive interactions among plant species and individuals.

## Graphical abstract

Using a farm‐scale experiment we investigated how intraspecific variation in flowering phenology and spatial heterogeneity in floral resources affected wild plant reproduction by modifying plant–pollinator interactions at the scale of the plant individual, the local assemblage, and the interaction network of agroecological infrastructures across the farm. We applied an original individual‐centered approach that ensured the spatio‐temporal variation in predictors of seed set (floral resources, pollinator activity, and interaction topology) matched exactly each individual plant's flowering period. These phenologically explicit models revealed that individual plant seed set in our two focal plant species (*Cyanus segetum* and *Centaurea jacea*) was influenced by patterns in plant–pollinator diversity and network structure that imply a co‐occurrence of pollinator‐mediated facilitative and competitive interactions operating within and between ecological scales. Icons sourced from: https://thenounproject.com/CC BY‐NC‐ND 2.0: (lluisa Iborra, Yu Luck, Les vieux garçons, Gan Khoon Lay, Blair Adams, Nareerat Jaikaew, and Prosymbols) and https://www.shutterstock.com (a free trial).

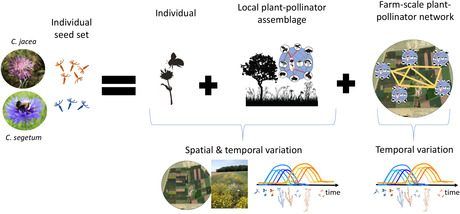

## Résumé

L’équilibre des relations de compétition et de facilitation entre plantes pour la pollinisation et la disponibilité des ressources abiotiques affectent le succès reproducteur des espèces et des individus de plantes. La succession temporelle et l’hétérogénéité spatiale des ressources florales modifient les interactions plantes‐pollinisateurs à différentes échelles écologiques (individu végétal, assemblage plantes‐pollinisateurs local, réseau d’interactions des infrastructures écologiques à travers la ferme). Les variations intraspécifiques de phénologie de floraison peuvent moduler le succès reproducteur individuel en déterminant le niveau d’hétérogénéité spatio‐temporelle de densité de donneurs de pollen, des interactions plantes‐pollinisateurs et des ressources florales auxquelles un individu de plante est exposé. Nous avons mené une expérimentation pour tester comment la production de graines des plantes sauvages est affectée par les interactions plantes‐pollinisateurs à différentes échelles écologiques, ces interactions étant modulées par la phénologie florale et l’hétérogénéité spatio‐temporelle des ressources florales (découlant des infrastructures agroécologiques). Nous avons transplanté 144 individus de deux espèces végétales entomophiles (*Cyanus segetum* et *Centaurea jacea*) dans des infrastructures agroécologiques (10 bandes fleuries et six bandes enherbées semées) d’un domaine agroécologique expérimental (125 ha). Ces espèces à phénologie de floraison contrastée présentent toutes deux une longue période de floraison avec des variations intraspécifiques qui déterminent l’exposition des individus aux interactions plantes‐pollinisateurs et aux conditions météorologiques. Nous avons appliqué une approche phénologiquement explicite centrée sur l’individu végétal, de manière à relier précisément la période de floraison de chaque individu aux niveaux correspondants d’hétérogénéité spatio‐temporelle des interactions plantes‐pollinisateurs, des densités de donneurs de pollen potentiels, des ressources florales (aux échelles de l’individu végétal, de l’assemblage local et du réseau d’interactions des infrastructures écologiques à travers la ferme) et des conditions abiotiques (température, précipitations, azote). L’attractivité individuelle (offre florale et taux de visite par les pollinisateurs) ainsi que la densité florale (toutes espèces) dans l’assemblage interspécifique local ont affecté positivement la production individuelle de graines des deux espèces végétales. Celle de *C. jacea* augmentait aussi directement avec la densité d’individus conspécifiques fleuris dans l’assemblage local. La densité de couplage du réseau a affecté positivement la production individuelle de graines des deux espèces et a influencé celle de *C. segetum* en modifiant l’effet de l’assemblage local (richesse et densité florales, richesse spécifique de pollinisateurs potentiels) sur le nombre de graines par individu. Le succès reproducteur individuel de *C. segetum* augmentait aussi avec le niveau de dépendance mutuelle entre l’espèce et ses pollinisateurs dans le réseau. Chez *C. jacea*, la production de graines individuelle était maximisée quand à la fois le niveau de spécialisation de *C. jacea* sur ses pollinisateurs dans le réseau et sa dépendance mutuelle à ses pollinisateurs étaient élevés. Les conditions abiotiques n’ont eu qu’un impact limité voire inexistant sur le succès reproducteur. Nos résultats montrent comment l’équilibre des interactions plantes‐pollinisateurs entre espèces et individus, peut répondre à l’hétérogénéité spatio‐temporelle liée à la gestion agroécologique de différentes façons qui affectent la reproduction des plantes sauvages. Les relations entre les interactions plantes‐pollinisateurs et la production individuelle de graines des plantes focales se déclinent entre et au sein de différentes échelles écologiques, de l’individu à la communauté, impliquant une co‐occurrence d’interactions facilitatrices et compétitrices entre espèces et individus de plantes via les pollinisateurs.

## 1 INTRODUCTION

Sexual reproduction in outcrossing plant species contributes to genetically diverse populations and reduces inbreeding risks (Eckert et al., 2010). Seed set is one component of plant reproductive success that results from the plant's physiological state and efficient conspecific pollen (= genes) transfer, which is facilitated by foraging insects in many flowering plant species (Ollerton et al., 2011). Physiological investment in reproduction by the plant individual (flowers and seeds) relies on the availability of water, energy, and nutrients, with any deficit likely to affect the physiological capacity for seed production (Akter & Klečka, 2022; Guilioni et al., 2003). Furthermore, intra‐ and interspecific plant competition for these abiotic resources can divert investment from sexual reproduction and modify the size of floral displays used to attract the pollinators needed for cross‐pollination (Akter & Klečka, 2022).

Plants may also compete for insect pollination because plant–pollinator interactions typically occur in multi‐species assemblages (Bascompte & Jordano, 2007). Indirect plant–plant interactions through pollinator sharing can reduce seed set due to interspecific pollen transfer that disrupts conspecific pollination (Arceo‐Gómez & Ashman, 2014; Morales & Traveset, 2008). Conversely, facilitation of plant reproductive success can also occur in diverse co‐flowering assemblages when greater pollinator densities or diversity increase flower visitation rates or provide complementarity and redundancy in pollination services (Blüthgen & Klein, 2011; Ghazoul, 2006; Hegland, 2014). Moreover, pollinator foraging behaviors vary with the relative quality, abundance, and accessibility of pollen or nectar due to the spatio‐temporal turnover in flowering plant assemblages (Gallagher & Campbell, 2020; Jha & Kremen, 2013; Lázaro et al., 2009). This combination of foraging plasticity and spatio‐temporal floral heterogeneity can determine the level of pollination competition or facilitation among plant individuals and species to alter plant reproductive outcomes.

This complexity of multispecies interactions and ecological processes influencing plant reproduction can be understood as a plant–pollinator network (Bascompte & Jordano, 2007). Variation in the organization and strength of species interactions in a network reflects community composition, species coevolution, and ecological processes like competition and resource partitioning (Junker et al., 2013; Magrach et al., 2021; Vázquez et al., 2009).

Greater network linkage density (encompassing the overall species richness and frequency of interactions) might affect pollination processes by increasing overall flower visitation rates (Akter et al., 2017) or by offering potential complementarity or redundancy in pollinators (Blüthgen & Klein, 2011; Ghazoul, 2006; Magrach et al., 2021; Venjakob et al., 2016). A high level of plant species specialization in pollinator interactions (*d′*—Blüthgen et al., 2006, 2008; Dormann et al., 2009) implies that pollen transfer relies on relatively few pollinator species in the multispecies network, which, under adaptive foraging, may dictate the potential for floral constancy, conspecific pollen transfer, and subsequent seed set (Valdovinos et al., 2013). This potential would be greatest where there is a strong mutual dependence between pollinator and plant species in the network re‐enforcing conspecific pollen transfer (Bascompte et al., 2006; Vázquez et al., 2007). However, an insect species dominating visitation to a particular plant species but with lower dependence because it forages on an array of forage plant species may dilute conspecific pollen deposition and interfere with plant reproduction through heterospecific pollen deposition (Arceo‐Gómez & Ashman, 2014; Morales & Traveset, 2008).

Consequently, variation in plant–pollinator network structure has the potential to indicate and influence pollination efficiency and plant reproduction, but relatively few studies have examined this relationship (Arceo‐Gómez et al., 2020; Arroyo‐Correa et al., 2021; Lázaro et al., 2020; Magrach et al., 2021; Theodorou et al., 2017; Vanbergen et al., 2014). Moreover, reports are idiosyncratic with neutral (Theodorou et al., 2017) or positive (Arroyo‐Correa et al., 2021; Lázaro et al., 2020) effects of network structure on seed production.

Plant–pollinator interactions are filtered by the combination of organism traits and environmental conditions at different levels of ecological organization (Arroyo‐Correa et al., 2021; Lázaro et al., 2009). Cost–benefit dynamics governing mutualistic plant–insect relationships (Bronstein, 1994) mean that reproductive outcomes are often unbalanced among plant species and individuals (Mesgaran et al., 2017). Furthermore, the occurrence of distinct assemblages of species interactions at different ecological scales (organism to community) may have complementary or opposing effects on plant pollination and reproduction (Hegland, 2014; cf. specialization—Brosi, 2016). For instance, plant species that are scarce within a species‐rich assemblage may experience dilution of pollinator visits, reduced pollen transfer, and seed production (Evans et al., 2017). Conversely, large intraspecific or interspecific floral displays can increase plant‐mating opportunities through the overall attraction of floral visitors (Akter et al., 2017; Hegland, 2014). However, a large floral display in an individual plant may increase the risk of geitonogamous pollen transfer (self‐pollination), which can increase risks of inbreeding and in self‐incompatible species reduce seed production (Akter et al., 2017; Eckert et al., 2010; Karron & Mitchell, 2012).

Apart from spatial effects, flowering phenology (i.e., the timing and duration of flowering period) is a species trait that influences plant reproduction. Inter‐ and intraspecific phenological variation filters the precise assemblage of interacting species that a plant individual is exposed to during the temporal succession of plants and pollinators, both in the immediate local assemblage and the wider community across the landscape (Arroyo‐Correa et al., 2021; CaraDonna & Waser, 2020; Gallagher & Campbell, 2020; Rafferty & Ives, 2012). The degree of overlap in the timing and duration of flowering periods, within and between species, therefore modulates the level of insect‐mediated conspecific pollen transfer and the balance of interspecific interactions (competition, interference, or facilitation) at different ecological scales affecting pollination services and plant seed set (Kovács‐Hostyánszki et al., 2013).

Pollinators and pollination services face anthropogenic threats with conventional intensive agricultural management the foremost worldwide (Dicks et al., 2021; Potts et al., 2016). Ecological intensification of agriculture is one alternative management model to reduce the negative impacts of agriculture and respond to global change while maintaining food production (Vanbergen et al., 2020). Ecologically intensive practices include the use and rotation of diverse crops and existing or restored agroecological infrastructures (e.g., semi‐natural habitats, sown wildflower, or grass strips) to harness ecosystem services, like pollination, in support of agriculture (Kovács‐Hostyánszki et al., 2017). Ecological intensification thus creates a heterogeneous and dynamic community in space and time, which is expected to modify pollinator interactions and wild plant reproductive success.

We aimed to understand how individual seed set in two wild plant species (*Cyanus segetum* Hill, and *Centaurea jacea* L.; Asteraceae) in herbaceous agroecological infrastructure was affected by spatio‐temporal heterogeneity in floral resources and plant–pollinator interactions across multiple ecological scales (individual—local assemblage—interaction network of agroecological infrastructure in the farm landscape). Because of the prolonged and variable flowering periods of each species (Monticelli et al., 2022), we used an individual‐centered and phenologically explicit approach to match precisely the flowering period of different plant individuals (transplanted into sown wildflower or grass–legume strips) to the concomitant level of spatio‐temporal heterogeneity in plant–pollinator interactions (at different ecological scales) and abiotic conditions. A priori this assumed that the intraspecific variation in flowering phenologies would modulate the level of seed set due to variable pollinator‐mediated competition or facilitation encountered by the plants in these spatio‐temporally distinct assemblages. We predicted that:

1. Individual seed set of focal plant species would be positively related to abiotic conditions (nitrogen, precipitation, and temperature) affecting the plant's physiological state and potential to invest in reproduction.
2. At the individual‐plant scale, higher seed production would be related to greater relative individual attractiveness to pollinators, defined as the combination of a larger relative individual floral display compared to the floral display of other conspecifics of the plot, and a greater associated pollinator visitation rate.
3. Seed set would be increased by greater conspecific pollen donor densities and by higher species richness of potential pollinators attracted by larger heterospecific blooms of flowers (species richness and densities) in the local assemblage surrounding the plot of focal conspecific individuals.
4. Greater linkage density (*Lq*) and specialization (*d′*) or mutual dependence (MD) of focal plants on their pollinators in the interaction network at the scale of agroecological infrastructure (sown wildflower and grass/legume strips) across the farm would increase seed set by providing connectivity of conspecific pollen transfer between spatially separated plants.
5. Seed set will be modulated by the interplay of plant–pollinator relationships occurring within and between ecological scales due to the mobility of insects transferring pollen.

## 2 MATERIAL AND METHODS

### 2.1 Focal plant species

*Cyanus segetum* Hill, 1762, and *Centaurea jacea* L., 1753 [Asteraceae], were chosen as phylogenetically related herbaceous species with contrasting flowering phenology and with populations on the study site. *C. segetum* is an annual segetal species with individuals flowering from May to July. *C. jacea* is a common perennial of grassy environments flowering in late summer (July–October in Burgundy, France; Tison & de Foucault, 2014). Both species require insect pollinators (*C. jacea*—self‐incompatible; *C. segetum*—pseudo‐self‐compatible) and provide high‐quality pollen and nectar resources for flower‐visiting insects (Bellanger et al., 2014; Hicks et al., 2016; Ouvrard et al., 2018; Steffan‐Dewenter et al., 2001). Their prolonged flowering periods (Monticelli et al., 2022) present intraspecific phenological variations that dictate individual plant exposure to plant–pollinator interactions.

### 2.2 Experimental design

The experiment was conducted (May–September 2019) on the INRAE CA‐SYS experimental farm (Burgundy, France, 47°19′06.7″N 5°04′17.6″E). Established in 2018, this farm‐scale agroecological system experiment (125 ha) is testing zero‐pesticide agroecological farming systems, including spatio‐temporal crop diversity and ecological infrastructures (Vanbergen et al., 2020). This included sowing 6.8 ha (22.67 km × 3 m) of grass–legume (seven species) strips and 2.69 ha (8.96 km × 3 m) of wildflower (37 species) strips with mixtures designed to promote pollination and biocontrol ecosystem services (Table S1). In mid‐March 2019, we transplanted 144 individuals per species, split into 16 plots of nine individuals per species (three triplets, 100 cm apart), located either in 10 wildflower strips or in six grass strips (together referred to as “agroecological infrastructure” hereafter), with plots at least 150 m apart (Figure S1). *C. segetum* focal individuals originated from ARBIOTECH (http://www.arbiotech.com) and were germinated in controlled conditions (22 ± 3°C; 16 h light: 8 h dark) prior to transplantation. We collected 48 large *C. jacea* rosettes (second‐year individuals ready to flower) from each of three local populations (CA‐SYS platform: 47°19′06.7″N 5°04′17.6″E; Dijon: 47°19′06.7″N 5°04′17.6″E; and Champdôtre: 47°10′42.5″N 5°17′02.0″E) and transplanted three individuals from each population into the experimental plots.

### 2.3 Abiotic environment‐influencing plant physiological capacity to invest in seed production

At the farm scale, we calculated the mean temperature (°C) and the mean precipitation (mm) for each individual's flowering period until harvested using daily records from an automated meteorological station at the experimental farm (Equations S1). At the individual plant scale, we used the foliar N content (%) of each plant individual as an indicator of its physiological state and a proxy for the biochemical resources available for investment in reproduction (Wang et al., 2018). One leaf sample per individual (~5 g) was collected before the flowering period of each species (*C. segetum*—mid‐May; *C. jacea*—early July). After oven drying (40°C) and milling (diameter ≤80 μm), the N content in 4–6 mg of foliar tissues (%) was quantified using a Thermo Scientific FLASH 2000 Organic Elemental Analyzer™.

### 2.4 Reproductive development and seed set of focal plant individuals

We counted the open and wilted composite flowers, floral buds, and fruits produced by each focal plant individual at monthly intervals. Combined with insect visitation data, these measurements allowed us to estimate the flowering period of each individual plant (precision to the week). At the end of the flowering period (i.e., *C. segetum*—mid‐July; *C. jacea*—early‐September), all surviving focal plants were harvested. We counted the total number of seeds produced per individual (hereafter “seed set”) and the total number of floral heads (fruits with or without seeds) as a measure of the size of the total individual floral display.

### 2.5 Local flowering plant assemblage

Centered on each focal plant plot, we established a transect (100 m × 2 m, *n* = 16 transects) to assess the local flowering plant assemblage and plant–pollinator interactions (see below). At monthly intervals, a team of two botanists surveyed the assemblages in six systematically placed quadrats (0.5 × 2 m) per transect to identify all entomophilous (Eudicotyledon) plant species present and the total number of floral units per species (a single or composite flower, spike, or umbel). From these data, we calculated corresponding to the flowering period of each individual focal plant: the mean floral richness (Equation S2), mean floral density (m^−2^; Equation S3), and the mean density (m^−2^) of potential conspecific pollen donors (*C. segetum* or *C. jacea*) in the local assemblage. Furthermore, to ensure representativity of the assemblages and interactions considered, these means were temporally weighted by the overlap between the individual plant's flowering period and the time period (number of days) covered by each botanical survey (Equations S2–S3).

### 2.6 Plant–pollinator interactions from individual to *local assemblage scales*

We quantified plant–pollinator interactions to each focal plant species (plot—Table S2) and in the local floral assemblage (transect—Tables S2 and S3) matched to focal individual flowering phenology (*C. segetum*: May–July; *C. jacea*: July–September, with only a brief phenological overlap in mid‐July). Bimonthly standardized sampling sessions were done per focal plant plot (15 min observing flowering ≤9 *C. segetum* or ≤9 *C. jacea* individuals) and per local floral community (15 min transect observing insects visiting all flowering species) with insect visitors actively feeding or having contact with floral stamen/anthers directly captured to avoid resampling individuals. Hymenoptera and Syrphidae were subsequently identified as species using standard keys (References S1), while other Diptera and a few Lepidoptera individuals were assigned to a Recognizable Taxonomic Unit (RTU = morphospecies approach following Oliver & Beattie, 1993).

Standard pollinator sampling protocols (09:30–17:30, in dry and warm weather ≥14°C and with minimal wind: Beaufort scale <4/5) were applied with each sampling round involving two teams of two people who, after an initial harmonization session, followed the identical protocol (insect capture and monitoring focal plant development) in parallel on different plots/transects. The order the plots/transects were sampled was randomized on each occasion (1–3 days for all plots/transects) to avoid introducing a systematic bias.

We quantified the relative attractiveness (*RIA*, *n* insect visits/individual/15 min) of each individual focal plant for foraging pollinators in terms of the visitation rate per 15 min weighted by the size of the individual floral display relative to the total plot‐level floral display of each focal plant species (Equation 1).

(1) $$\begin{array}{c} {\mathrm{RIA}}_{i}=\sum_{s=1}^{N}\frac{n\;\text{visits on focal conspecific flowers of the}\;{\text{plot}}_{s}*n\;{\text{flowers}}_{i,s}}{n\;\text{tot conspecific focal flowers in the}\;{\text{plot}}_{s}}/N \end{array}$$

where *i* = the focal individual considered; *s* = a single pollinator survey conducted on the plot of conspecific focals including individual *i* during the flowering period of individual *i*; *N* = the total number of pollinator surveys conducted during the flowering period of individual *i*.

We also calculated the local species richness of potential pollinators of *C. segetum* or *C. jacea* during each individual's flowering period. We defined potential pollinators as insects observed visiting focal or non‐focal *C. segetum* or *C. jacea*, respectively, in the surveyed agroecological infrastructure (Table S2). For each focal plant individual, only those insect species captured foraging (on any plant species) in the local assemblage surrounding the individual during its flowering period were included in the species list of potential pollinators.

### 2.7 Structure of plant–pollinator networks of agroecological infrastructure across the farm

To create phenologically explicit quantitative (weighted) bipartite networks matching the flowering period of each focal plant individual, we summed the data of plant–pollinator interactions across the assemblages in the farm (16 transects) and the focal plots of *C. segetum* and *C. jacea* (16 plots) collected during each individual's flowering period.

We calculated linkage density *Lq*—the mean number of links per species weighted by the mean frequency of interactions (Dormann et al., 2009). This indicates the wider diversity and density of plant–pollinator interactions in the agroecological infrastructure across the farm and hence the potential activity and species redundancy in the pollination service to the focal plants. The potential level of conspecific pollen transfer in the network was described using two network metrics: plant species specialization in pollinator interactions (*d′*—Blüthgen et al., 2006) and total weighted mutual dependence between the focal plant species and its pollinators (MD; Equation 2—following Bascompte et al., 2006). While *d′* describes the diversity of focal plant interactions, MD accounts for pollinator constancy in terms of the relative amount of pollinator interactions on the focal plants compared to other plant species in the network.

(2) $${\mathrm{MD}}_{c}=\sum_{p=1}^{N}\left(\frac{n\;{\text{interactions}}_{p,c}-1}{\text{total}\;n\;{\text{interactions}}_{c}-1}*\frac{n\;{\text{interactions}}_{p,c}-1}{\text{total}\;n\;{\text{interactions}}_{p}-1}\right)$$

where *c* = the focal plant species considered (*C. segetum* or *C. jacea* here); *p* = a pollinator species having visited the focal plant species (*C. segetum* or *C. jacea*); *N* = the total number of pollinator species having visited the focal plant species (*C. segetum* or *C. jacea*).

The dependence of the plant species (c) on pollinator p and reciprocally (p on c) were multiplied and the products were summed across all pollinator species to give the total mutual dependence of each focal plant species. The interactions were weighted according to the total observation frequency of pollinator species to exclude pollinator species only observed once (Blüthgen et al., 2008). This index varies between 0 (weak mutual dependence) and 1 (strong mutual dependence = pairwise mutualism or perfect nestedness), with higher values expected to reflect increased efficiency of focal plant pollen transfer (Vázquez et al., 2007).

### 2.8 Statistical analyses

We used one generalized linear mixed model per species (GLM, “lme4”) to explain the intraspecific variations in seed set between surviving focal individuals of *C. segetum* (*n* = 144) and *C. jacea* (*n* = 105) fitting a negative binomial distribution to control for overdispersion. We fitted “plot” as a random effect to account for the spatial dispersion of the replicates and unmeasured microsite conditions (singularity meant we dropped this random effect in the *C. jacea* model to avoid overfitting).

Predictors of seed set reflecting the influence of the abiotic environment on each individual plant's growth and reproductive capacity were the foliar N content (%), mean temperature (°C), and mean precipitation (mm). Spatio‐temporal heterogeneity in plant–pollinator interactions and floral assemblages at different ecological scales (focal plant individual; local assemblage; and interaction network of agroecological infrastructure across the farm) were fitted as fixed effects and two‐way interactions between and within scales. These fixed effects predicted to affect seed set were the relative attractiveness of the focal plant (*n* visits/individual/15 min) at the individual plant scale and, at the local assemblage scale, the floral species richness, floral density (m^−2^), density of potential conspecific pollen donors (m^−2^), and local species richness of potential pollinators. At the scale of the interaction network of agroecological infrastructure, we fitted the network linkage density (*Lq*), focal plant specialization (*d′*), and the total weighted MD between the focal plant species and its pollinators.

All predictors were calculated to correspond to the individual flowering period of the 249 focal plants that produced a fruit (≥1). This ensured high precision in the estimation of the abiotic context and assemblages of plant–pollinator interactions the individual was exposed to at the level of the individual plant, local assemblage, and the interaction network of agroecological infrastructure across the farm (Table S4).

From each full model, we used an AIC‐based multi‐model selection procedure (ΔAIC <2; package MuMIn), avoiding the input of highly correlated variables (>70% Pearson coefficient), to identify the best subset of seed set predictors for each species according to their relative sum of weights (ω_i_) contained in the top‐ranked models. For *C. jacea*, to obtain model convergence, we had to compute several selection steps dropping certain predictors in turn from full models (foliar N content, density of potential conspecific pollen donors, individual attractiveness × floral richness or floral density, and floral density or floral richness × species richness of potential pollinators). Then, we ran a final selection process on the most complete full model containing parameters previously retained in the successive selection steps, and retained the best average model and subset of predictors.

Predictors were scaled (*z*‐transformation) and log‐transformed where necessary to improve model fit. Model assumptions (normality and homoscedasticity of residuals) were checked (package DHARMa), as was collinearity among model predictors (package performance). We used the “effects” and “ggplot2” packages to calculate and visualize the marginal effects (fitted lines) of the predictors in both models. All statistical procedures were performed with R Studio (version 4.2.0).

## 3 RESULTS

*C. segetum* and *C. jacea* individuals that reached the flowering stage produced a mean (± SE) of 250.1 ± 29.4 seeds and 980.8 ± 188.5 seeds, respectively. In total, 1034 insects (Hymenoptera = 840 individuals, Diptera = 188, and Lepidoptera = 6) from 90 species were captured foraging on flowers of focal plant species (*C. segetum*: 197 individual insects; *C. jacea*: 86 individual insects) and the wider floral assemblages (751 individual insects; for species abundances and identities, see Tables S2 and S3). Including non‐focal conspecifics in the plant assemblages (transects), the number of potential pollinators observed foraging on *C. segetum* and *C. jacea* were, respectively, 356 insects from 26 species and 88 insects from 24 species (Table S2).

### 3.1 Abiotic variables influencing focal plant seed set

Only mean temperature over the flowering period related negatively and positively to seed set in *C. segetum* and *C. jacea*, respectively (Tables 1 and 2). Neither precipitation nor foliar N content were selected in the best models.

**TABLE 1:** Final GLMM of *C. segetum* seed set in response to abiotic (N, H_2_O, and °C) and biotic (plant–pollinator interactions and floral assemblage) predictors matched to the flowering period of focal plant individuals at the scale of the individual plant, local assemblage, and the plant–pollinator network of the agroecological infrastructure across the farm.

Ecological scale	Predictor	*β* ± SE	*z* value	*p*‐value
	Intercept	4.32 ± 0.26	16.75	<.001
Abiotic	Temperature (°C)	−0.19 ± 0.10	−1.89	<.1
Individual focal plant	Relative individual attractiveness (*RIA*) (log)	0.86 ± 0.08	10.44	<.001
Local assemblage	Species richness of potential pollinators	−0.002 ± 0.21	−0.009	>.1
	Floral richness	−0.07 ± 0.14	−0.51	>.1
	Floral density (log)	0.60 ± 0.23	2.59	<.01
	Species richness of potential pollinators × floral richness	0.20 ± 0.13	1.48	>.1
Network of agroecological infrastructure	Linkage density (*Lq*) (log)	0.46 ± 0.11	4.17	<.001
	Mutual dependence (MD)	0.24 ± 0.08	3.06	<.01
Network × local assemblage	Species richness of potential pollinators × linkage density (log)	0.56 ± 0.15	3.77	<.001
	Floral richness (log) × linkage density (log)	−0.77 ± 0.21	−3.71	<.001
	Floral density (log) × linkage density (log)	−0.14 ± 0.05	−2.93	<.01

*Note*: The GLMM (negative binomial) was derived from AIC‐based multi‐model selection, and predictors were present in >50% of the 22 best models (ΔAIC <2). Total variance explained (*R* ^2^) by marginal (fixed effects) and conditional (fixed + random) predictors, and the plot random effect (σ^2^) are cited. Predictors were log‐transformed where required to account for non‐linear relationships with the log of seed number.

**Model fit**: *R* ^2^ marginal = 0.65; *R* ^2^ conditional = 0.93; random σ^2^ = 0.95.

**TABLE 2:** Final GLM of *C. jacea* seed set in response to abiotic (N, H_2_O, and °C) and biotic (plant–pollinator interactions and floral assemblage) predictors matched to the flowering period of focal plant individuals at the scale of the individual plant, local assemblage, and the plant–pollinator network of the agroecological infrastructure across the farm.

Ecological scale	Parameter selected in the best model	*β* ± SE	*z* value	*p*‐value
	Intercept	5.26 ± 0.13	40.58	<.001
Abiotic	Temperature	0.79 ± 0.40	2.00	<.05
Individual focal plant	Relative individual attractiveness (*RIA*) (log)	1.94 ± 0.23	8.38	<.001
Local assemblage	Species richness of potential pollinators	0.16 ± 0.15	1.07	>.1
	Density of potential pollen donors (log)	0.31 ± 0.11	2.84	<.01
	Floral density	−0.27 ± 0.13	−2.15	<.05
Local assemblage × individual focal plant	Floral density × relative individual attractiveness (log)	0.84 ± 0.20	4.29	<.001
Network of agroecological infrastructure	Linkage density (*Lq*) (log)	0.70 ± 0.40	1.74	<.1
	*C. jacea* specialization (*d′*) (log)	−0.63 ± 0.30	−2.09	<.05
	Mutual dependence (MD)	0.34 ± 0.25	1.37	>.1
	*C. jacea* specialization (log) × mutual dependence	0.42 ± 0.24	3.03	<.01

*Note*: The GLM (negative binomial) was derived from AIC‐based multi‐model selection and predictors were present in >60% of the 15 best models (ΔAIC <2). The random effect was dropped due to model singularity and to avoid overfitting; therefore, the total variance explained (*R* ^2^) is due solely to the fixed effects. Predictors were log‐transformed (*n* + 0.0001) where required to account for non‐linear relationships with the log of seed number.

**Model fit:** *R* ^2^ = 0.79.

### 3.2 Plant–pollinator interactions influencing seed set at focal plant and local assemblage scales

**FIGURE 1:**
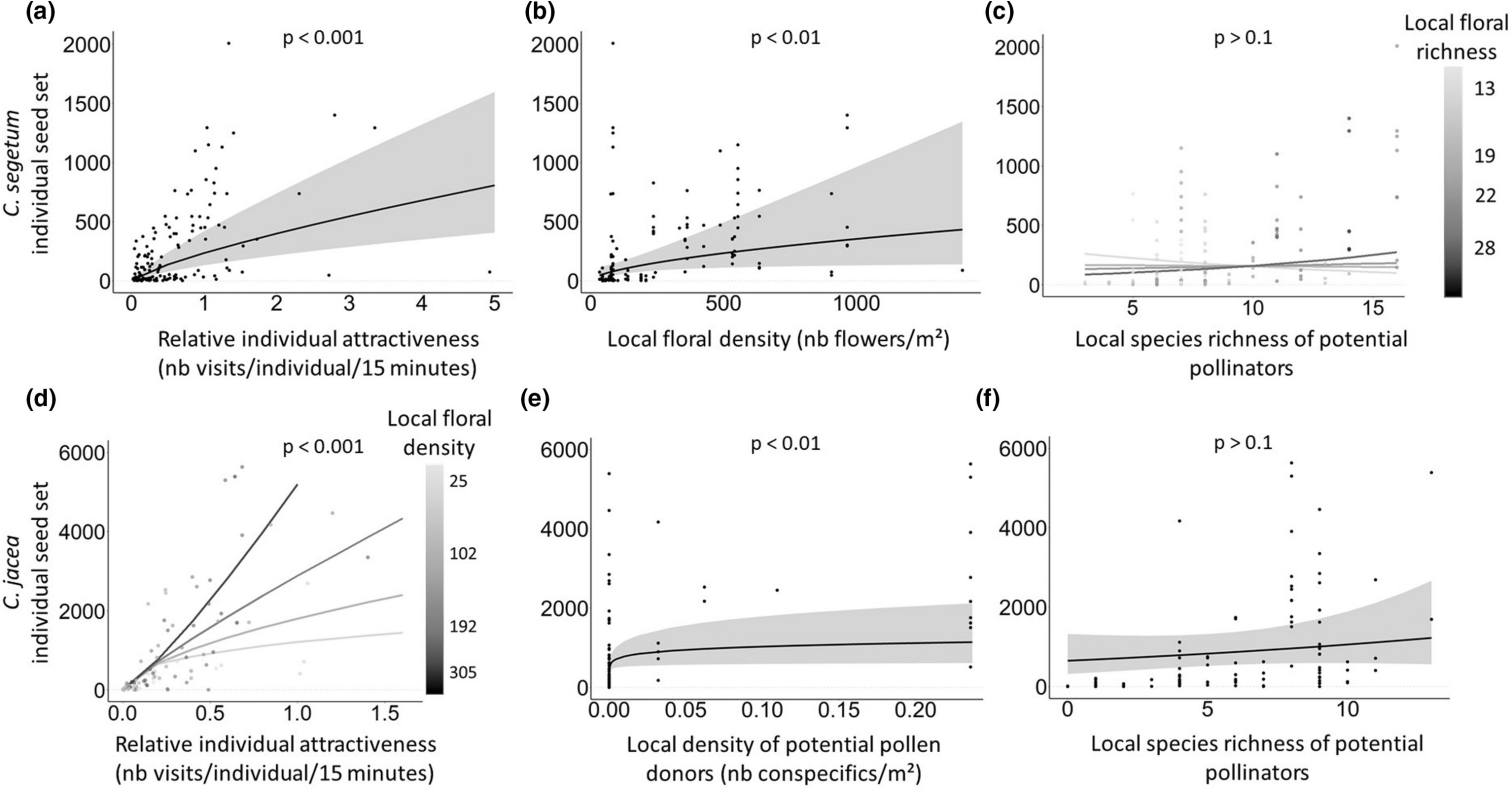
Seed yield of focal *C. segetum* (a–c) and *C. jacea* (d–f) individuals in relation to: (a) relative individual attractiveness; (b) assemblage floral density; (c) assemblage floral richness × species richness of potential pollinators; (d) relative individual attractiveness × assemblage floral density; (e) local density of potential pollen donors; and (f) species richness of potential pollinators. Fitted lines (± CI) are partial residuals accounting for other fixed and random (*C. segetum*) effects from GLMMs. To visualize the interactive effect on seed set, values of local floral density and richness were fixed to the mean of the data included in each of the four data quarters, using the three quartiles as threshold values. The scatter plots show the distribution of the raw data. One point (*C. jacea* seed set = 15,769) was removed from graphs d, e, and f for better visualization (but not from the calculation of marginal effects).

The seed set of *C. segetum* and *C. jacea* was positively related to the relative attractiveness of each individual focal plant for foraging pollinators (Tables 1 and 2; Figure 1a,d) and the floral density in the local plant assemblage (Tables 1 and 2; Figure 1b,d). Moreover, for *C. jacea*, this relationship between seed set and the relative individual attractiveness was accentuated by increasing floral density (Table 2; Figure 1d). *C. jacea* seed set also responded positively to the density of potential conspecific pollen donors in the local assemblage (Table 2; Figure 1e), but *C. segetum* was unaffected.

Although for both focal plant species, the species richness of potential pollinators active in the local floral assemblage was among the main fixed effects predicting seed set, its effect was relatively weak compared to other parameters (Tables 1 and 2; Figure 1c,f). An albeit weak statistical interaction (floral richness × potential pollinator richness) indicated that the response of *C. segetum* seed set to the species richness of potential pollinators foraging in the local assemblage was negative when situated in florally poor local assemblages, but positive in most species‐rich floral assemblages (Table 1; Figure 1c).

### 3.3 Properties of plant–pollinator network structure in agroecological infrastructure affecting seed set

**FIGURE 2:**
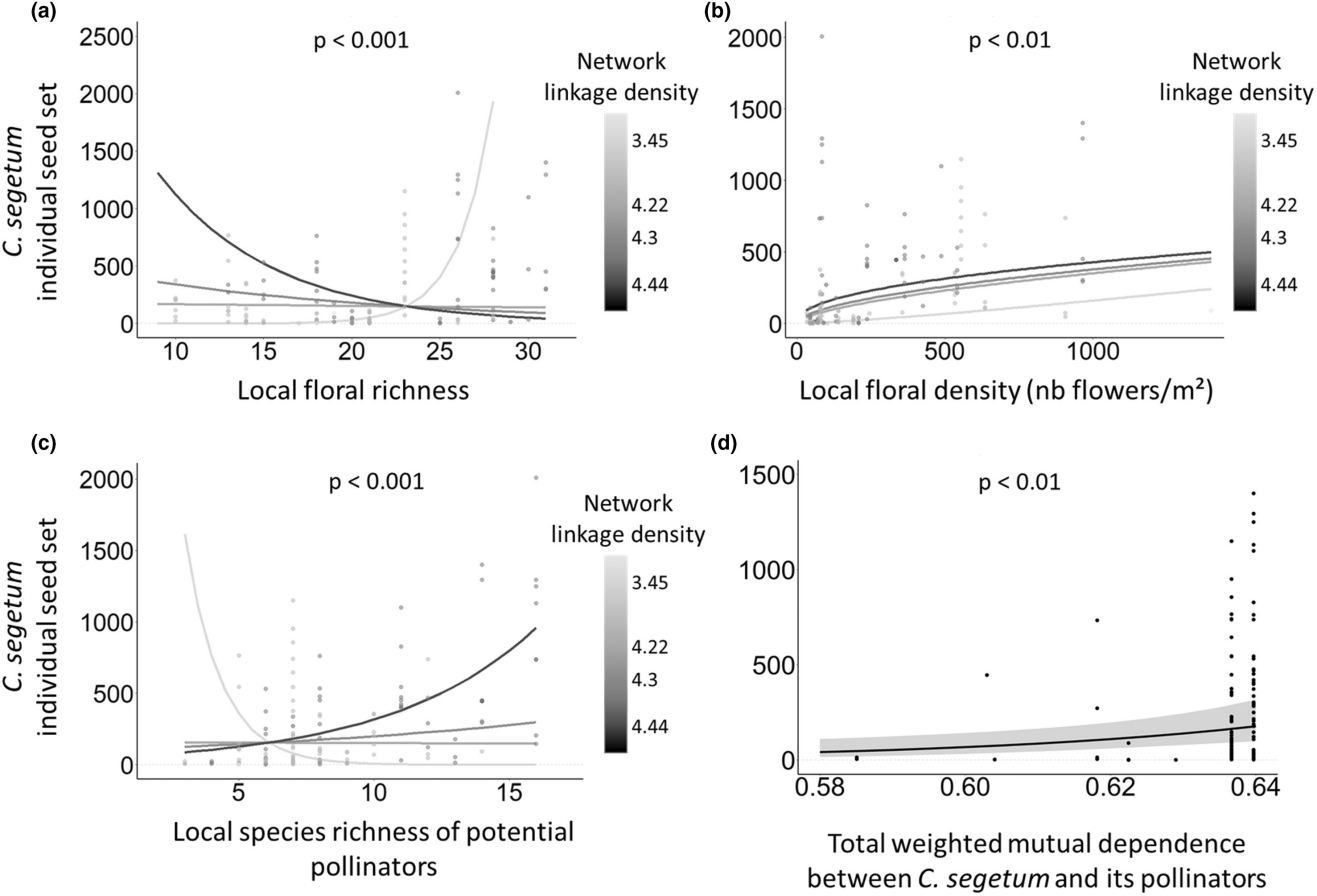
Seed yield of focal *C. segetum* individuals in relation to: Network linkage density interacting with (a) assemblage floral richness, (b) assemblage floral density, and (c) species richness of potential pollinators; and (d) the mutual dependence between *C. segetum* and its pollinators. Fitted lines (± CI) are partial residuals from GLMMs accounting for other fixed and random effects. The scatter plots show the distribution of the raw data. One point was deleted from graph *d* (seed set = 2009) for better visualization (but not from the calculation of marginal effects).

**FIGURE 3:**
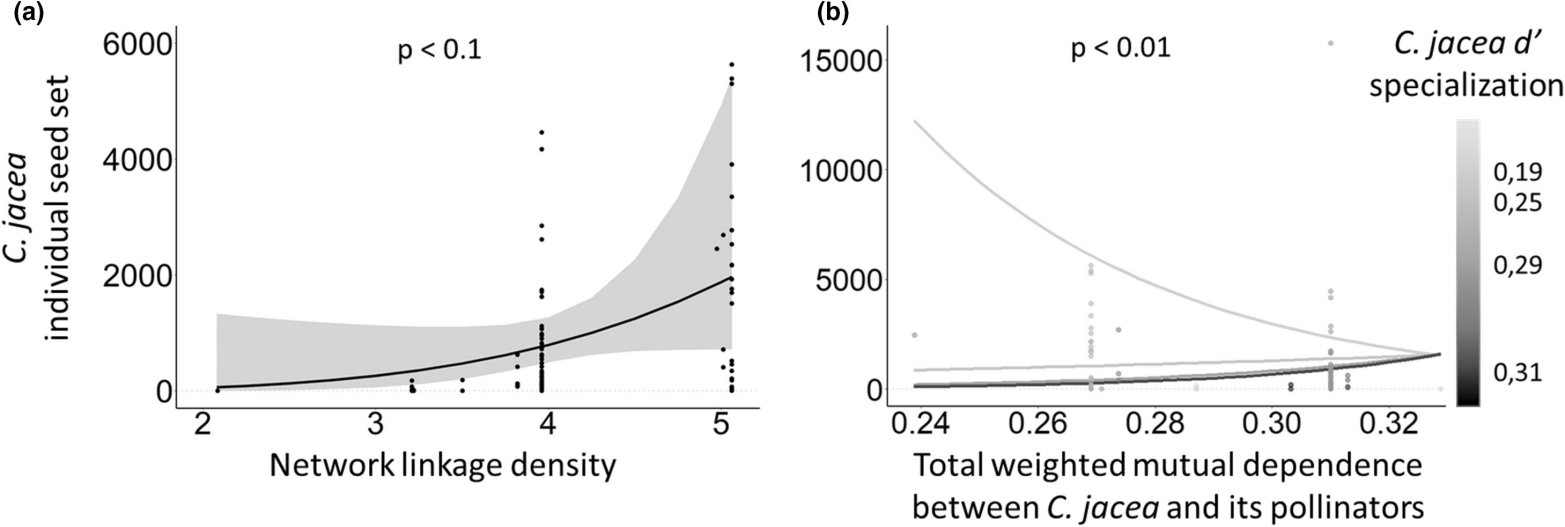
Seed yield of focal *C. jacea* individuals in relation to (a) network linkage density and (b) *d′* specialization × mutual dependence between *C. jacea* and its pollinators. Fitted lines (± CI) are partial residuals from GLMs accounting for other fixed effects. The scatter plots show the distribution of the raw data. One point was deleted from graph A (seed set = 15,769) for better visualization (but not from the calculation of marginal effects).

As a main effect, network linkage density positively affected seed set in both focal species (Tables 1 and 2; Figures 2a–c and 3a). Network linkage density also influenced *C. segetum* reproduction by modifying the effect on individual seed set of the density and species richness of the local floral assemblage or richness of potential pollinators foraging locally (Table 1; Figure 2a–c).

*C. segetum* seed set was negatively related to greater floral richness in the local assemblage when network linkage density was higher and only responded positively to local floral richness under lowest values of network linkage density (Table 1; Figure 2a). The strength of the positive relationship between local floral density and *C. segetum* seed set increased as the network linkage density increased (Table 1; Figure 2b). In the more densely linked networks, the local species richness of potential pollinators had a positive relation to *C. segetum* seed set, with only a negative relationship at the lowest level of linkage density (Table 1; Figure 2c).

*C. jacea* specialization in pollinator interactions (*d′*) had a negative impact on its individual seed set as a main fixed effect (Table 2), but *C. segetum* was unaffected. As a main fixed effect, the effect of total weighted MD between *C. segetum* and its pollinators had a positive influence on seed set (Table 1; Figure 2d). For *C. jacea*, there was a strong interaction (*d′* × MD) showing a positive effect on seed set as both specialization and mutual dependence increased, but a negative effect on seed set under low values of *d′* (Table 2; Figure 3b).

Overall, the final best model including environmental temperature and variation in floral assemblages and plant–pollinator interactions across ecological scales explained (*R* ^2^) a high level of the phenologically explicit variation in individual seed production of *C. segetum* and *C. jacea* (Tables 1 and 2).

## 4 DISCUSSION

Our individual‐based, phenologically explicit approach and models accounted for the intraspecific variation in flowering phenology that can affect pollination processes by filtering the precise combination of spatio‐temporal heterogeneity of floral resources and plant–pollinator interactions to which the individual plant was exposed (Arroyo‐Correa et al., 2021; CaraDonna & Waser, 2020; Rafferty & Ives, 2012). As predicted, focal plant reproduction was affected by the combination of pollinator interactions and floral resource heterogeneity operating at the scale of the plant individual, the local assemblage, and the plant–pollinator network of agroecological infrastructure across the farm landscape (Arroyo‐Correa et al., 2021; Hegland, 2014; Kovács‐Hostyánszki et al., 2013).

Nitrogen and water availability were predicted to affect the plant's capacity to invest in reproduction (Akter & Klečka, 2022; Guilioni et al., 2003), but they were unimportant in this case, perhaps because the farm‐scale (125 ha) environmental gradients in abiotic resources were insufficiently strong to affect seed production. Although the environmental temperature at the farm scale was selected in the final model for both species, it was generally a less important determinant of seed production than floral assemblages or plant–pollinator interactions.

The size of the individual floral display is an important determinant of insect visitation rate, pollination, and seed set (Akter et al., 2017; Karron & Mitchell, 2012). As predicted, seed set for both focal species was related positively to the individual plant's relative attractiveness, an index integrating the plant's capacity to invest in a large floral display, the attraction via the floral display of conspecific neighbors, and the corresponding pollinator visitation rate (Akter et al., 2017). Individual *C. jacea* with larger floral displays also had greater success at attracting and concentrating visitation with subsequent benefits for seed set when situated within assemblages with greater interspecific floral densities. Along with the direct relationship between focal plant seed set and floral density in the local assemblage, this suggests the overall attraction of pollinators to a dense floral community (Hegland, 2014) whose plastic foraging behaviors then facilitated the transfer of pollen among focal plants (Jha & Kremen, 2013; Petanidou et al., 2008).

A greater density of potential conspecific pollen donors in the local assemblage enhanced individual seed set of *C. jacea* but not *C. segetum*. This difference is possibly due to the relative population size of the two focal species (Table S4). Although both focal species were part of the sown wildflower seed mix, *C. jacea* only flowers after a year of vegetative growth, whereas *C. segetum* is annual (Nitschke et al., 2010; Tison & de Foucault, 2014). Consequently, the availability of potential pollen donors was more limiting for *C. jacea* with only few naturally occurring individuals on the farm providing a source of outcross pollen in addition to the transplanted focal individuals, whereas in the sown mixtures there were readily available pollen donors for *C. segetum* (Eckert et al., 2010).

The interplay between pollinator and floral species richness in the local assemblage further affected *C. segetum* reproduction, although relatively weakly, compared to other predictors. *C. segetum* seed set tended to decrease or increase with increasing local species richness of potential pollinators when assemblage floral richness was low or high, respectively. A potential explanation is that in species‐poor floral assemblages, although plant competition for pollinators was reduced (Arceo‐Gómez & Ashman, 2014), there was greater interspecific competition and interference among pollinator species enhancing their movement (Fontaine et al., 2008; Greenleaf & Kremen, 2006) in ways that disrupted conspecific pollen transfer and lowered seed set. Alternatively, where species richness of the floral assemblage and foraging pollinators were both higher, niche partitioning may have reduced such competitive interactions (Brosi & Briggs, 2013), thereby improving floral constancy and facilitating conspecific pollen transfer and *C. segetum* seed set (Morales & Traveset, 2008). If correct, this interpretation shows how the balance of interspecific competition (plant and pollinator; Arceo‐Gómez & Ashman, 2014; Fontaine et al., 2008; Hegland, 2014) and foraging plasticity of pollinators via niche partitioning in local assemblages (Jha & Kremen, 2013; Valdovinos et al., 2013) can influence plant species reproductive outcomes.

The structure of plant–pollinator networks in agroecological infrastructure across the farm also influenced the reproduction of the individual focal plants during their respective flowering periods. Linkage density provides a metric of the overall species richness and frequency of interactions in the network (Dormann et al., 2009). Greater network linkage density had a strong positive influence on individual *C. segetum* seed set (but less strongly for *C. jacea*). Furthermore, the species richness of potential pollinators and floral density in the local assemblage most benefited *C. segetum* seed set when linkage density was higher, showing how spatio‐temporal heterogeneity across ecological scales influenced plant reproduction (Hegland, 2014; Kovács‐Hostyánszki et al., 2013). Together, these results suggest that the reproduction of these focal species (particularly *C. segetum*) visited mostly by generalist pollinators (Table S2) benefited from being embedded within a wider species‐rich network with high flower visitation rates. This might be due to adaptive foraging (Valdovinos et al., 2013), trait matching (Garibaldi et al., 2015), and/or species complementarity or redundancy (Blüthgen & Klein, 2011; Venjakob et al., 2016; Woodcock et al., 2019) in the pollination service, which may have diluted plant competition for pollinators.

In contrast, local floral richness had a positive influence on *C. segetum* seed set only under low values of linkage density. This effect became negative as observed network linkage density increased. This implies that at low levels of linkage density (and potential complementarity or redundancy) the higher levels of floral species richness in the local assemblage concentrate pollinator activity (Ghazoul, 2006; Jha & Kremen, 2013; Potts et al., 2009), spilling over to benefit *C. segetum* pollination and reproduction. However, an increase in linkage density at the farm scale may have enhanced interspecific pollinator‐mediated plant competition at the local scale, hindering efficient conspecific pollen transfer (Arceo‐Gómez & Ashman, 2014; Morales & Traveset, 2008).

We also predicted that seed set would be modulated by the level of focal plant specialization on pollinators (*d′*) or MD in the farm‐scale network of agroecological infrastructure through gains in conspecific pollen transfer between spatially separated plants (Bascompte et al., 2006; Valdovinos et al., 2013; Vázquez et al., 2007). A greater level of MD between the focal species and their pollinators, indicating higher constancy of pollinator interactions on the focal plants compared to other plant species, contributed to increase seed set (Morales & Traveset, 2008; Vázquez et al., 2007). For *C. jacea*, the positive effect of MD was augmented when specialization on pollinators (*d′*) was high. Pollinator networks are typically nested, meaning there is a high reliance of specialist plants on generalist pollinators foraging on many floral species (Bascompte & Jordano, 2007), potentially diluting conspecific pollen transfer (Arceo‐Gómez et al., 2020; Lázaro et al., 2020). Under adaptive foraging, those generalist pollinators may minimize competition by concentrating on their specialist plant partners (Valdovinos et al., 2013). Concomitantly high levels of MD and *d′* may reflect a level of adaptive foraging that overcame the negative influence of nestedness on conspecific pollen transfer. Whereas when *C. jacea* specialization was low, there was an inverse relationship between MD and seed set which may be consistent with interspecific pollinator interferences resulting from high pollinator activity on *C. jacea* (Greenleaf & Kremen, 2006).

Our analysis reveals the complex species‐specific patterns in wild plant visitation and seed set driven by the distribution of floral resources and pollinator foraging movements in a heterogeneous farmed landscape (Jha & Kremen, 2013; Kovács‐Hostyánszki et al., 2013). Overall, *C. jacea* seed set was supported by the attraction of pollinators to individual floral displays, by interspecific floral densities in the neighboring assemblage, and pollinator constancy of visits to conspecifics in the network of agroecological infrastructure across the farm (Hegland, 2014; Morales & Traveset, 2008). *C. segetum* individual seed set benefited from a more complex combination of intra‐ and interspecific biodiversity and interactions operating at individual plant, local assemblage, and wider network levels (Arroyo‐Correa et al., 2021; Hegland, 2014). These differences reflected the seasonal turnover in floral resources distributed across the agroecological infrastructure and the dynamics and co‐occurrence of pollination facilitation and competition operating within and between ecological scales (Brosi, 2016; Hegland, 2014; Kovács‐Hostyánszki et al., 2013; Mesgaran et al., 2017).

A caveat to our study is that we only detect correlative patterns in pollinator and plant biodiversity and interactions, which we interpret according to known ecological processes (e.g., competition vs facilitation). Although we predicted and controlled for the effect of intra‐ and interspecific phenology on plant–pollinator interactions, the two plant species differ in other traits (e.g., life cycle; Tison & de Foucault, 2014) that may also affect their interaction with pollinators. Additional field experiments that manipulate these processes (e.g., competition and temporal turnover) or assemblage structure (e.g., diversity, trait, or functional group structure) are needed to verify the precise mechanisms that produced the observed patterns at different ecological scales (Magrach et al., 2021).

Our results highlight how the balance of intra‐ and interspecific plant–pollinator interactions may respond to spatio‐temporal heterogeneity arising from individual phenology and agroecological management, in ways that affect wild plant species reproduction. How these biotic interactions are affecting the longer‐term population persistence and genetic health of these wild flowering plant species remains unknown, however, but has implications for the performance of agroecological farm management in terms of weed management and provision of biodiversity and ecosystem services.

## AUTHOR CONTRIBUTIONS

**Lucie S. Monticelli:** Methodology (supporting); validation (supporting); visualization (supporting); writing – review and editing (supporting). **Mélinda Turpin:** Investigation (supporting); methodology (equal); validation (supporting); visualization (supporting); writing – review and editing (supporting). **Emeline Felten:** Investigation (supporting); methodology (equal); validation (supporting); writing – review and editing (supporting). **Emilien Laurent:** Investigation (supporting); methodology (equal); validation (supporting); writing – review and editing (supporting). **Annick Matejicek:** Investigation (supporting); methodology (equal). **Luc Biju‐Duval:** Methodology (equal). **Chantal Ducourtieux:** Methodology (equal). **Eric Vieren:** Methodology (equal). **Violaine Deytieux:** Conceptualization (equal); methodology (supporting); resources (equal). **Stéphane Cordeau:** Conceptualization (equal); methodology (supporting); writing – original draft (supporting); writing – review and editing (supporting). **David Bohan:** Formal analysis (supporting); validation (supporting); visualization (supporting); writing – review and editing (supporting). **Adam J. Vanbergen:** Conceptualization (lead); formal analysis (supporting); funding acquisition (lead); investigation (equal); methodology (equal); project administration (lead); supervision (lead); validation (equal); writing – original draft (equal); writing – review and editing (equal).

## FUNDING INFORMATION

ANER Bourgogne‐Franche‐Comté (ESREA Project), Grant/Award Number: C2325‐1552‐1565; European Commission (H2020 Safeguard), Grant/Award Number: GA 101003476; French Ministry for Education and Research (Ecole Doctorale Environnement‐Santé, Université de Bourgogne), Grant/Award Number: MESR 2019‐18; Institut National de Recherche pour l'Agriculture, l'Alimentation et l'Environnement (SPE ‐ SCOURGE Project), Grant/Award Number: INRAE‐SPE‐IB2019.

## CONFLICT OF INTEREST

The authors declare no conflict of interest.

## Supporting information


Appendix S1

## Data Availability

Data are available via the Dryad Digital Repository: doi: 10.5061/dryad.bvq83bkdk.
