# Supplementary material for: Individual flowering phenology shapes plant–pollinator interactions across ecological scales affecting plant reproduction
Source: Ecol Evol. 2023 Jan 4;13(1):e9707. doi: 10.1002/ece3.9707 (PMC9811238; doi:10.1002/ece3.9707)
Supplement: Supplementary file 1 — Appendix S1 [file ECE3-13-e9707-s001.doc]

# Supplementary material

**Table S1**. Composition of wildflower and grass/legume mixture sown in the field borders across the INRAE CA-SYS experimental farm (Burgundy, France) and realized flowering species richness observed during the floral and pollinator surveys.

| **Sown species** | | | |
| --- | --- | --- | --- |
| **Wildflower strips** | | **Grass strips** | |
| *Achillea millefolium* | *Leucanthemum vulgare* | *Dactylis glomerata* |  |
| *Alliaria petiolata* | *Malva sylvestris* | *Festuca arundinacea* |  |
| *Barbarea vulgaris* | *Medicago sativa* | *Festuca pratensis* |  |
| *Bellis perennis* | *Melilotus officinalis* | *Festuca rubra* |  |
| *Capsella bursa-pastoris* | *Onobrychis viciifolia* | *Lotus corniculatus* |  |
| *Centaurea jacea* | *Origanum vulgare* | *Trifolium repens* |  |
| *Centaurea scabiosa* | *Papaver rhoeas* | *Vicia sativa* |  |
| *Cichorium intybus* | *Pastinaca sativa* |  |  |
| *Cota tinctoria* | *Pimpinella saxifraga* |  |  |
| *Cyanus segetum* | *Plantago lanceolata* |  |  |
| *Daucus carota* | *Ranunculus acris* |  |  |
| *Echium vulgare* | *Securigera varia* |  |  |
| *Foeniculum vulgare* | *Stellaria media* |  |  |
| *Galium mollugo* | *Tanacetum vulgare* |  |  |
| *Geum urbanum* | *Taraxacum officinale* |  |  |
| *Hesperis matronalis* | *Veronica persica* |  |  |
| *Hypericum perforatum* | *Vicia sativa* |  |  |
| *Jacobaea vulgaris* | *Vicia sepium* |  |  |
| *Knautia arvensis* |  |  |  |
|  |  |  |  |
| **Observed flowering species** | | | |
| **Wildflower strips** | | **Grass strips** | |
| *Achillea millefolium* | *Lathyrus sativus* | *Apiaceae sp.* | *Papaver dubium* |
| *Aethusa cynapium* | *Lepidium didymum* | *Brassica napus* | *Papaver rhoeas* |
| *Asteraceae sp.* | *Leucanthemum vulgare* | *Capsella bursa-pastoris* | *Papaver sp.* |
| *Bellis perennis* | *Linum usitatissimum* | *Chenopodium album* | *Persicaria lapathifolia* |
| *Brassica napus* | *Lotus corniculatus* | *Cirsium arvense* | *Persicaria maculosa* |
| *Capsella bursa-pastoris* | *Lysimachia arvensis* | *Cirsium vulgare* | *Picris hieracioides* |
| *Centaurea horticole* | *Malva sp.* | *Convolvulus arvensis* | *Polygonum aviculare* |
| *Centaurea jacea* | *Matricaria sp.* | *Daucus carota* | *Senecio inaequidens* |
| *Centaurea scabiosa* | *Medicago lupulina* | *Euphorbia exigua* | *Senecio vulgaris* |
| *Centaurea sp.* | *Medicago sativa* | *Euphorbia helioscopia* | *Silene latifolia* |
| *Cerastium sp.* | *Melilotus officinalis* | *Fallopia convolvulus* | *Sinapis alba* |
| *Chaenorrhinum minus* | *Onobrychis viciifolia* | *Fumaria officinalis* | *Sinapis arvensis* |
| *Chenopodium album* | *Papaver rhoeas* | *Galium aparine* | *Sinapis sp.* |
| *Cichorium intybus* | *Papaver sp.* | *Helianthus sp.* | *Stellaria media* |
| *Cirsium arvense* | *Persicaria lapathifolia* | *Helminthotheca echioides* | *Trifolium repens* |
| *Cirsium sp.* | *Persicaria maculosa* | *Kickxia spuria* | *Tripleurospermum inodorum* |
| *Cirsium vulgare* | *Picris hieracioides* | *Lactuca serriola* | *Veronica arvensis* |
| *Convolvulus arvensis* | *Plantago lanceolata* | *Lysimachia arvensis* | *Veronica hederifolia* |
| *Cota tinctoria* | *Polygonum aviculare* | *Matricaria sp.* | *Veronica persica* |
| *Cyanus segetum* | *Senecio vulgaris* | *Medicago lupulina* | *Vicia faba* |
| *Daucus carota* | *Sinapis arvensis* | *Medicago sativa* | *Viola arvensis* |
| *Echium vulgare* | *Sinapis sp.* |  |  |
| *Euphorbia exigua* | *Sonchus asper* |  |  |
| *Euphorbia helioscopia* | *Stellaria media* |  |  |
| *Fallopia convolvulus* | *Thlaspi arvense* |  |  |
| *Foeniculum vulgare* | *Torilis arvensis* |  |  |
| *Fumaria officinalis* | *Trifolium repens* |  |  |
| *Galium aparine* | *Tripleurospermum inodorum* |  |  |
| *Geranium colombinum* | *Veronica arvensis* |  |  |
| *Geranium dissectum* | *Veronica hederifolia* |  |  |
| *Geranium pusillum* | *Veronica persica* |  |  |
| *Helminthotheca echioides* | *Vicia faba* |  |  |
| *Kickxia spuria* | *Vicia sativa* |  |  |
| *Lactuca serriola* | *Viola arvensis* |  |  |

**Table S2**. Identity, abundance, and mutual dependence of the insect pollinator species observed visiting focal or non-focal *C. segetum* (black), *C. jacea* (grey) and on both species (bold) on the INRAE CA-SYS experimental farm (Burgundy, France) in spring-summer 2019. Note: The total abundance of an insect species over the whole experiment duration is often less than the sum of the visits to all plant species during the respective flowering periods of the focal plants (*C. segetum* and *C. jacea*). This is because there was an overlap (2 weeks in July) in the focal species flowering period so that individuals of certain insect species shared between the focal plant species were recorded on the transects while both focal *C. segetum* and focal *C. jacea* in the plot were flowering, causing them to appear twice in the tabulated summary below. Parentheses () indicate insect species present in the field margins during the focal plant flowering, but that were only recorded foraging on plant species other than the focal plant species. RTU are morphospecies defined following Oliver & Beattie (1993).

| Order | Family | Insect species | Insect abundance during *C. segetum* flowering period | | Mutual dependence of insect species and *C. segetum* during its flowering period | | Insect abundance during *C. jacea* flowering period | | | Mutual dependence of insect species and *C. jacea* during its flowering period | | **Total insect abundance over the experiment duration** |
| --- | --- | --- | --- | --- | --- | --- | --- | --- | --- | --- | --- | --- |
|  |  |  | All plant species | *C. segetum* only | n visits to *C. segetum* /N visits to all plant species (%) | n visits *to C. segetum* / total N visitors to *C. segetum* (%) | All plant species | *C. jacea* only | n visits to *C. jacea* /N visits to all plant species (%) | | n visits to *C. jacea* / total N visitors to *C. jacea* (%) |  |
| HYMENOPTERA | Andrenidae | ***Andrena flavipes*** | **17** | **11** | **65%** | **3.1%** | **20** | **3** | **15%** | | **3.4%** | **22** |
|  | Apidae | ***Apis mellifera*** | **146** | **126** | **86%** | **35.4%** | **90** | **29** | **32%** | | **33.0%** | **211** |
|  |  | ***Bombus lapidarius*** | **38** | **32** | **84%** | **9.0%** | **32** | **6** | **19%** | | **6.8%** | **50** |
|  |  | *B. pascuorum* | 4 | 1 | 25% | 0.3% | (5) |  |  | |  | 8 |
|  |  | *B. ruderarius* | 1 | 1 | 100% | 0.3% | (1) |  |  | |  | 1 |
|  |  | ***B. sylvarum*** | **3** | **2** | **67%** | 0.6% | **10** | **1** | **10%** | | **1.1%** | **10** |
|  |  | ***B. terrestris/lucorum*** | **30** | **13** | **43%** | **3.7%** | **20** | **3** | **15%** | | **3.4%** | **43** |
|  |  | *Ceratina cucurbitina* | 1 | 1 | 100% | **0.3%** |  |  |  | |  | 1 |
|  |  | *Ceratina cyanea* |  |  |  |  | 1 | 1 | 100% | | 1.1% | 1 |

| Order | Family | Insect species | Insect abundance during *C. segetum* flowering period | | Mutual dependence of insect species and *C. segetum* during its flowering period | | | Insect abundance during *C. jacea* flowering period | | Mutual dependence of insect species and *C. jacea* during its flowering period | | **Total insect abundance over the experiment duration** |
| --- | --- | --- | --- | --- | --- | --- | --- | --- | --- | --- | --- | --- |
|  |  |  | All plant species | *C. segetum* only | n visits to *C. segetum* /N visits to all plant species (%) | | n visits *to C. segetum* / total N visitors to *C. segetum* (%) | All plant species | *C. jacea* only | n visits to *C. jacea* /N visits to all plant species (%) | n visits to *C. jacea* / total N visitors to *C. jacea* (%) |  |
| HYMENOPTERA | Halictidae | ***Halictus cf. simplex*** | **8** | **5** | **63%** | **1.4%** | | **10** | **7** | **70%** | **8.0%** | **14** |
|  |  | ***H. scabiosae*** | **17** | **13** | **76%** | **3.7%** | | **13** | **5** | **38%** | **5.7%** | **27** |
|  |  | *H. smaragdulus* |  |  |  |  | | 1 | 1 | 100% | 1.1% | 1 |
|  |  | *H. subauratus* | (1) |  |  |  | | 2 | 1 | 50% | 1.1% | 2 |
|  |  | *H. tumulorum* | 1 | 1 | 100% | 0.3% | | (2) |  |  |  | 2 |
|  |  | ***Lasioglossum glabriusculum*** | **35** | **3** | **9%** | **0.8%** | | **36** | **4** | **11%** | **4.5%** | **42** |
|  |  | *L. interruptum* | 7 | 6 | 86% | 1.7% | | (7) |  |  |  | 8 |
|  |  | ***L. leucozonium*** | **2** | **1** | **50%** | **0.3%** | | **2** | **1** | **50%** | **1.1%** | **4** |
|  |  | ***L. malachurum*** | **117** | **60** | **51%** | **16.9%** | | **53** | **9** | **17%** | **10.2%** | **130** |
|  |  | *L. nigripes* | 1 | 1 | 100% | 0.3% | |  |  |  |  | 1 |
|  |  | ***L. pauxillum*** | **44** | **7** | **16%** | **2.0%** | | **53** | **2** | **4%** | **2.3%** | **72** |
|  |  | *L. politum* | (7) |  |  |  | | 6 | 1 | 17% | 1.1% | 12 |
|  |  | *L. punctatissimum* |  |  |  |  | | 1 | 1 | 100% | 1.1% | 1 |
|  |  | *L. puncticolle* | 3 | 1 | 33% | 0.3% | | (2) |  |  |  | 4 |
|  |  | *L. subhirtum* | 4 | 2 | 50% | 0.6% | | (6) |  |  |  | 6 |
|  |  | *L. villosulum* | (8) |  |  |  | | 53 | 4 | 8% | 4.5% | 57 |
|  | Megachilidae | *Megachile pilidens* |  |  |  |  | | 2 | 1 | 50% | 1.1% | 2 |
|  | Vespidae | *Polistes dominulus* | 1 | 1 | 100% | 0.3% | | (4) |  |  |  | 5 |
| **Total hymenoptera** |  |  | **480** | **288** | **60%** | **80.9%** | | **404** | **80** | **20%** | **90.9%** | **737** |

| Order | Family | Insect species | Insect abundance during *C. segetum* flowering period | | Mutual dependence of insect species and *C. segetum* during its flowering period | | Insect abundance during *C. jacea* flowering period | | Mutual dependence of insect species and *C. jacea* during its flowering period | | **Total insect abundance over the experiment duration** |
| --- | --- | --- | --- | --- | --- | --- | --- | --- | --- | --- | --- |
|  |  |  | All plant species | *C. segetum* only | n visits to *C. segetum* /N visits to all plant species (%) | n visits *to C. segetum* / total N visitors to *C. segetum* (%) | All plant species | *C. jacea* only | n visits to *C. jacea* /N visits to all plant species (%) | n visits to *C. jacea* / total N visitors to *C. jacea* (%) |  |
| Diptera | Syrphidae | ***Episyrphus balteatus*** | **63** | **16** | **25%** | **4.5%** | **2** | **1** | **50%** | **1.1%** | **65** |
|  |  | *Eupeodes corollae* | 18 | 14 | 78% | 3.9% |  |  |  |  | 19 |
|  |  | *Scaeva pyrastri* | 6 | 4 | 67% | 1.1% |  |  |  |  | 6 |
|  |  | *Sphaerophoria scripta* | 48 | 32 | 67% | 9.0% |  |  |  |  | 57 |
|  |  | RTUd1 | 1 | 1 | 100% | 0.3% | (2) |  |  |  | 3 |
|  | Other families | RTUd11 |  |  |  |  | 3 | 2 | 67% | 2.3% | 3 |
|  |  | RTUd15 |  |  |  |  | 1 | 1 | 100% | 1.1% | 1 |
|  |  | RTUd22 |  |  |  |  | 1 | 1 | 100% | 1.1% | 1 |
|  |  | RTUd7 | (1) |  |  |  | 3 | 2 | 67% | 2.3% | 3 |
|  |  | RTUd9 |  |  |  |  | 1 | 1 | 100% | 1.1% | 1 |
| **TOTAL DIPTERA** |  |  | **136** | **67** | **49%** | **18.8%** | **11** | **8** | **73%** | **9.1%** | **159** |
| Lepidoptera |  | RTUl2 | 1 | 1 | 100% | 0.3% |  |  |  |  | 1 |
| **TOTAL LEPIDOPTERA** |  |  | **1** | **1** | **1** | **0.3%** |  |  |  |  | **1** |
| **TOTAL** |  |  | **617** | **356** | **57.7%** | **100%** | **416** | **88** | **21.2%** | **1%** | **897** |

**Table S3.** Abundance of insect species active on the study site during the experiment but not interacting with *C. segetum* nor with *C. jacea*. The abundances are given respectively during *C. segetum* (black) and during *C. jacea* (grey) flowering period, and in total over the whole experiment duration (bold).

| Order | Family | Insect species | Insect abundance during *C. segetum* flowering period | Insect abundance during *C. jacea* flowering period | **Total insect abundance over the experiment duration** |
| --- | --- | --- | --- | --- | --- |
| HYMENOPTERA | Andrenidae | *Andrena chrysosceles* | 2 | 0 | **2** |
|  |  | *Andrena distinguenda* | 1 | 0 | **1** |
|  |  | *Andrena labiate* | 1 | 0 | **1** |
|  |  | *Andrena lagopus* | 1 | 0 | **1** |
|  |  | *Andrena minutula* | 1 | 1 | **1** |
|  |  | *Andrena nana* | 4 | 5 | **5** |
|  |  | *Andrena nigroaena* | 1 | 0 | **1** |
|  |  | *Andrena nitidiuscula* | 13 | 13 | **13** |
|  |  | *Andrena semilaevis* | 1 | 1 | **1** |
|  | Apidae | *Bombus hortorum* | 5 | 0 | **5** |
|  |  | *Nomada_sp.1* | 0 | 2 | **2** |
|  | Halictidae | *Dufourea sp.* | 0 | 1 | **1** |
|  |  | *Halictus maculatus* | 2 | 2 | **3** |
|  |  | *Lasioglossum laticeps* | 7 | 0 | **7** |
|  |  | *Lasioglossum morio* | 1 | 0 | **1** |
|  |  | *Sphecodes crassus* | 0 | 1 | **1** |
|  |  | *Sphecodes gibbus* | 1 | 1 | **1** |
|  |  | *Sphecodes monilicornis* | 2 | 2 | **2** |
|  |  | *Sphecodes_sp.1* | 0 | 2 | **2** |
|  | Megachilidae | *Megachile maritima* | 0 | 1 | **1** |
|  | Cephidae | *Cephus_sp.1* | 44 | 0 | **44** |
|  |  | *Cephus_sp.2* | 2 | 0 | **2** |
|  |  | *Monoplopus_sp.1* | 1 | 0 | **1** |
|  | Vespidae | *Eumenes pomiformis* | 0 | 1 | **1** |
|  |  | *Polistes bischoffi* | 0 | 1 | **1** |
|  |  | *Polistes gallicus* | 0 | 2 | **2** |
| **Total hymenoptera** |  |  | 90 | 36 | **103** |

| Order | Family | Insect species | Abundance during *C. segetum* flowering period | Abundance during *C. jacea* flowering period | **Total abundance over the experiment duration** |
| --- | --- | --- | --- | --- | --- |
| DIPTERA | Syrphidae | *Eristalis arbustorum* | 1 | 1 | **1** |
|  |  | *Paragus_constrictus/*  *tibialis* | 1 | 1 | **1** |
|  |  | *Paragus haemorrhous* | 2 | 4 | **4** |
|  |  | *Syritta pipiens* | 0 | 1 | **1** |
|  |  | *Syrphus vitripennis* | 1 | 0 | **1** |
|  | Other families | RTUd2 | 1 | 1 | **1** |
|  |  | RTUd3 | 1 | 1 | **1** |
|  |  | RTUd4 | 1 | 1 | **1** |
|  |  | RTUd5 | 1 | 1 | **1** |
|  |  | RTUd6 | 1 | 1 | **1** |
|  |  | RTUd8 | 1 | 1 | **1** |
|  |  | RTUd10 | 1 | 1 | **1** |
|  |  | RTUd13 | 0 | 1 | **1** |
|  |  | RTUd14 | 0 | 1 | **1** |
|  |  | RTUd16 | 2 | 5 | **5** |
|  |  | RTUd17 | 1 | 2 | **2** |
|  |  | RTUd18 | 1 | 1 | **1** |
|  |  | RTUd20 | 0 | 1 | **1** |
|  |  | RTUd23 | 1 | 0 | **1** |
|  |  | RTUd24 | 0 | 1 | **1** |
|  |  | RTUd25 | 1 | 1 | **1** |
| **TOTAL DIPTERA** |  |  | 17 | 27 | **29** |
| LEPIDOPTERA |  | *Polyommatus icarus* | 1 | 0 | **1** |
|  | Diverse families | RTUl1 | 1 | 0 | **1** |
|  |  | RTUl3 | 1 | 0 | **1** |
|  |  | RTUl5 | 1 | 1 | **1** |
|  |  | RTUl7 | 1 | 1 | **1** |
| **TOTAL LEPIDOPTERA** |  |  | 5 | 2 | **5** |
| **TOTAL** |  |  | 112 | 65 | **137** |

**Table S4.** Mean values (± SD) of the candidate predictors of *C. segetum* and *C. jacea* seed set in the full models that vary according to the phenological period of focal plant flowering (date ranges of groups of individuals) and spatial position on field borders on the farm (F= wild flowers; G =grass-legume).

|  | ***C. segetum*** | | | | ***C. jacea*** | | |
| --- | --- | --- | --- | --- | --- | --- | --- |
| **Predictor** | **Flowering period** | **Nb of individuals** | | **Mean ± SD** | **Flowering period** | **Nb of individuals** | **Mean ± SD** |
| Temperature (°C) | 12/05 - 02/07 | | 3 | 18.48 ± 0.00 | 07/07 - 13/08 | 1 | 21.50 |
|  | 12/05 - 16/07 | | 1 | 18.59 | 07/07 - 01/09 | 3 | 20.72 ± 0.00 |
|  | 20/05 - 02/07 | | 2 | 19.47 ± 0.00 | 07/07 - 11/09 | 4 | 20.73 ± 0.02 |
|  | 20/05 - 16/07 | | 7 | 19.62 ± 0.32 | 21/07 - 13/08 | 2 | 21.23 ± 0.15 |
|  | 29/05 - 11/07 | | 1 | 20.35 | 21/07 - 01/09 | 6 | 20.52 ± 0.44 |
|  | 29/05 - 16/07 | | 51 | 20.36 ± 0.02 | 21/07 - 11/09 | 73 | 20.52 ± 0.13 |
|  | 11/06 - 02/07 | | 3 | 21.70 ± 0.20 | 13/08 - 01/09 | 5 | 19.05 ± 0.04 |
|  | 11/06 - 16/07 | | 76 | 21.44 ± 0.44 | 13/08 - 11/09 | 6 | 19.06 ± 0.04 |
|  |  | |  |  | 02/09 - 11/09 | 5 | 17.48 ± 0.04 |
|  | **TOTAL**  ***C. segetum*** | | **144** | **20.85 ± 0.80** | **TOTAL**  ***C. jacea*** | **105** | **20.26 ± 0.91** |
| Precipitation (mm) | 12/05 - 02/07 | | 3 | 0.88 ± 0.00 | 07/07 - 13/08 | 1 | 1.66 |
|  | 12/05 - 16/07 | | 1 | 0.82 | 07/07 - 01/09 | 3 | 1.35 ± 0.00 |
|  | 20/05 - 02/07 | | 2 | 0.98 ± 0.00 | 07/07 - 11/09 | 4 | 1.26 ± 0.01 |
|  | 20/05 - 16/07 | | 7 | 0.98 ± 0.07 | 21/07 - 13/08 | 2 | 2.59 ± 0.06 |
|  | 29/05 - 11/07 | | 1 | 1.13 | 21/07 - 01/09 | 6 | 1.69 ± 0.11 |
|  | 29/05 - 16/07 | | 51 | 1.06 ± 0.05 | 21/07 - 11/09 | 73 | 1.61 ± 0.04 |
|  | 11/06 - 02/07 | | 3 | 1.16 ± 0.08 | 13/08 - 01/09 | 5 | 0.59 ± 0.01 |
|  | 11/06 - 16/07 | | 76 | 1.07 ± 0.12 | 13/08 - 11/09 | 6 | 0.59 ± 0.01 |
|  |  | |  |  | 02/09 - 11/09 | 5 | 1.12 ± 0.57 |
|  | **TOTAL**  ***C. segetum*** | | **144** | **1.06 ± 0.10** | **TOTAL**  ***C. jacea*** | **105** | **1.48 ± 0.38** |

|  | ***C. segetum*** | | | ***C. jacea*** | | |
| --- | --- | --- | --- | --- | --- | --- |
| **Predictor** | **Flowering period** | **Nb of individuals** | **Mean ± SD** | **Flowering period** | **Nb of individuals** | **Mean ± SD** |
| Network linkage density | 12/05 - 02/07 | 3 | 4.27 ± 0.00 | 07/07 - 13/08 | 1 | 4.97 |
|  | 12/05 - 16/07 | 1 | 4.80 | 10/07 - 01/09 | 3 | 5.01 ± 0.00 |
|  | 20/05 - 11/07 | 2 | 3.74 ± 0.00 | 07/07 - 11/09 | 4 | 5.06 ± 0.00 |
|  | 20/05 - 16/07 | 7 | 4.39 ± 0.00 | 21/07 - 13/08 | 2 | 3.50 ± 0.00 |
|  | 29/05 - 11/07 | 1 | 3.54 | 21/07 - 01/09 | 6 | 3.82 ± 0.00 |
|  | 29/05 - 16/07 | 51 | 4.31 ± 0.00 | 21/07 - 11/09 | 73 | 4.25 ± 0.49 |
|  | 29/05 - 11/07 | 3 | 3.23 ± 0.00 | 13/08 - 01/09 | 5 | 3.22 ± 0.00 |
|  | 11/06 - 16/07 | 76 | 4.22 ± 0.00 | 13/08 - 11/09 | 6 | 3.21 ± 0.00 |
|  |  |  |  | 02/09 - 11/09 | 5 | 2.91 ± 1.30 |
|  | **TOTAL**  ***C. segetum*** | **144** | **4.23 ± 0.18** | **TOTAL**  ***C. jacea*** | **105** | **4.10 ± 0.69** |
| Species-level d’ specialization | 12/05 - 02/07 | 3 | 0.34 ± 0.00 | 07/07 - 13/08 | 1 | 0.30 |
|  | 12/05 - 16/07 | 1 | 0.37 | 10/07 - 01/09 | 3 | 0.30 ± 0.00 |
|  | 20/05 - 11/07 | 2 | 0.30 ± 0.00 | 07/07 - 11/09 | 4 | 0.29 ± 0.00 |
|  | 20/05 - 16/07 | 7 | 0.36 ± 0.00 | 21/07 - 13/08 | 2 | 0.36 ± 0.00 |
|  | 29/05 - 11/07 | 1 | 0.23 | 21/07 - 01/09 | 6 | 0.31 ± 0.00 |
|  | 29/05 - 16/07 | 51 | 0.36 ± 0.00 | 21/07 - 11/09 | 73 | 0.30 ± 0.00 |
|  | 29/05 - 11/07 | 3 | 0.18 ± 0.00 | 13/08 - 01/09 | 5 | 0.16 ± 0.00 |
|  | 11/06 - 16/07 | 76 | 0.36 ± 0.00 | 13/08 - 11/09 | 6 | 0.18 ± 0.00 |
|  |  |  |  | 02/09 - 11/09 | 5 | 0.26 ± 0.05 |
|  | **TOTAL**  ***C. segetum*** | **144** | **0.35 ± 0.03** | **TOTAL**  ***C. jacea*** | **105** | **0.28 ± 0.04** |
| Total standardized mutual dependence | 12/05 - 02/07 | 3 | 0.59 ± 0.00 | 07/07 - 13/08 | 1 | 0.24 |
|  | 12/05 - 16/07 | 1 | 0.60 | 10/07 - 01/09 | 3 | 0.27 ± 0.00 |
|  | 20/05 - 11/07 | 2 | 0.60 ± 0.00 | 07/07 - 11/09 | 4 | 0.27 ± 0.00 |
|  | 20/05 - 16/07 | 7 | 0.62 ± 0.00 | 21/07 - 13/08 | 2 | 0.30 ± 0.00 |
|  | 29/05 - 11/07 | 1 | 0.63 | 21/07 - 01/09 | 6 | 0.31 ± 0.00 |
|  | 29/05 - 16/07 | 51 | 0.64 ± 0.00 | 21/07 - 11/09 | 73 | 0.30 ± 0.02 |
|  | 29/05 - 11/07 | 3 | 0.62 ± 0.00 | 13/08 - 01/09 | 5 | 0.33 ± 0.00 |
|  | 11/06 - 16/07 | 76 | 0.64 ± 0.00 | 13/08 - 11/09 | 6 | 0.29 ± 0.00 |
|  |  |  |  | 02/09 - 11/09 | 5 | 0.27 ± 0.01 |
|  | **TOTAL**  ***C. segetum*** | **144** | **0.63 ± 0.01** | **TOTAL**  ***C. jacea*** | **105** | **0.30 ± 0.02** |
| Relative individual attractiveness (insect visits/individual/15 minutes) | 12/05 - 02/07 | 3 | 0.26 ± 0.21 | 07/07 - 13/08 | 1 | 0.25 |
|  | 12/05 - 16/07 | 1 | 0.30 | 10/07 - 01/09 | 3 | 1.04 ± 0.02 |
|  | 20/05 - 11/07 | 2 | 0.18 ± 0.20 | 07/07 - 11/09 | 4 | 0.42 ± 0.26 |
|  | 20/05 - 16/07 | 7 | 0.27 ± 0.30 | 21/07 - 13/08 | 2 | 0.18 ± 0.26 |
|  | 29/05 - 11/07 | 1 | 0.14 | 21/07 - 01/09 | 6 | 0.14 ± 0.21 |
|  | 29/05 - 16/07 | 51 | 0.74 ± 0.65 | 21/07 - 11/09 | 73 | 0.29 ± 0.31 |
|  | 29/05 - 11/07 | 3 | 0.25 ± 0.11 | 13/08 - 01/09 | 5 | 0.02 ± 0.03 |
|  | 11/06 - 16/07 | 76 | 0.56 ± 0.73 | 13/08 - 11/09 | 6 | 0.02 ± 0.04 |
|  |  |  |  | 02/09 - 11/09 | 5 | 0.01 ± 0.03 |
|  | **TOTAL**  ***C. segetum*** | **144** | **0.59 ± 0.67** | **TOTAL**  ***C. jacea*** | **105** | **0.27 ± 0.32** |

|  | ***C. segetum*** | | | | ***C. jacea*** | | | | |
| --- | --- | --- | --- | --- | --- | --- | --- | --- | --- |
| **Predictor** | **Flowering period** | **Nb of individuals** | | **Mean ± SD** | **Flowering period** | **Nb of individuals** | | **Mean ± SD** | |
| Local species richness of potential pollinators | 12/05 - 02/07 | 3 | | 8.00 ± 0.00 | 07/07 - 13/08 | 1 | | 8.00 | |
|  | 12/05 - 16/07 | 1 | | 14.00 | 10/07 - 01/09 | 3 | | 11.00 ± 0.00 | |
|  | 20/05 - 11/07 | 2 | | 6.50 ± 2.12 | 07/07 - 11/09 | 4 | | 10.50 ± 2.89 | |
|  | 20/05 - 16/07 | 7 | | 8.14 ± 3.72 | 21/07 - 13/08 | 2 | | 3.50 ± 4.95 | |
|  | 29/05 - 11/07 | 1 | | 7.00 | 21/07 - 01/09 | 6 | | 8.33 ± 2.66 | |
|  | 29/05 - 16/07 | 51 | | 10.08 ± 3.79 | 21/07 - 11/09 | 73 | | 6.33 ± 2.43 | |
|  | 29/05 - 11/07 | 3 | | 5.33 ± 0.58 | 13/08 - 01/09 | 5 | | 1.20 ± 1.30 | |
|  | 11/06 - 16/07 | 76 | | 7.25 ± 2.09 | 13/08 - 11/09 | 6 | | 1.50 ± 0.84 | |
|  |  |  | |  | 02/09 - 11/09 | 5 | | 1.40 ± 0.55 | |
|  | **TOTAL**  ***C. segetum*** | **144** | | **8.31 ± 3.18** | **TOTAL**  ***C. jacea*** | **105** | | **5.94 ± 3.22** | |
| Local floral richness | 12/05 - 02/07 | 3 | | 20.00 ± 0.00 | 07/07 - 13/08 | 1 | | 15.00 | |
|  | 12/05 - 16/07 | 1 | | 28.00 | 10/07 - 01/09 | 3 | | 5.00 ± 0.00 | |
|  | 20/05 - 11/07 | 2 | | 19.00 ± 2.83 | 07/07 - 11/09 | 4 | | 17.00 ± 2.31 | |
|  | 20/05 - 16/07 | 7 | | 21.86 ± 5.37 | 21/07 - 13/08 | 2 | | 10.50 ± 4.95 | |
|  | 29/05 - 11/07 | 1 | | 14.00 | 21/07 - 01/09 | 6 | | 8.50 ± 5.43 | |
|  | 29/05 - 16/07 | 51 | | 23.51 ± 5.43 | 21/07 - 11/09 | 73 | | 13.45 ± 4.81 | |
|  | 29/05 - 11/07 | 3 | | 18.33 ± 0.58 | 13/08 - 01/09 | 5 | | 4.40 ± 3.21 | |
|  | 11/06 - 16/07 | 76 | | 18.03 ± 4.96 | 13/08 - 11/09 | 6 | | 5.67 ± 3.14 | |
|  |  |  | |  | 02/09 - 11/09 | 5 | | 8.20 ± 4.55 | |
|  | **TOTAL**  ***C. segetum*** | **144** | | **20.26 ± 5.64** | **TOTAL**  ***C. jacea*** | **105** | | **11.90 ± 5.47** | |
| Local floral density (/m²) | 12/05 - 02/07 | | 3 | 68.64 ± 0.00 | 07/07 - 13/08 | | 1 | | 104.66 |
|  | 12/05 - 16/07 | | 1 | 334.18 | 10/07 - 01/09 | | 3 | | 44.30 ± 0.00 |
|  | 20/05 - 11/07 | | 2 | 79.59 ± 32.37 | 07/07 - 11/09 | | 4 | | 232.00 ± 113.88 |
|  | 20/05 - 16/07 | | 7 | 93.95 ± 52.39 | 21/07 - 13/08 | | 2 | | 392.09 ± 176.41 |
|  | 29/05 - 11/07 | | 1 | 83.00 | 21/07 - 01/09 | | 6 | | 53.96 ± 41.46 |
|  | 29/05 - 16/07 | | 51 | 301.03 ± 271.79 | 21/07 - 11/09 | | 73 | | 169.15 ± 108.09 |
|  | 29/05 - 11/07 | | 3 | 496.13 ± 781.99 | 13/08 - 01/09 | | 5 | | 82.9 ± 75.22 |
|  | 11/06 - 16/07 | | 76 | 241.86 ± 246.64 | 13/08 - 11/09 | | 6 | | 105.75 ± 50.73 |
|  |  | |  |  | 02/09 - 11/09 | | 5 | | 84.61 ± 105.45 |
|  | **TOTAL**  ***C. segetum*** | | **144** | **254.60 ± 266.75** | **TOTAL**  ***C. jacea*** | | **105** | | **153.27 ± 112.49** |
| Local density of potential pollen donors (/m²) | 12/05 - 02/07 | | 3 | 0.00 ± 0.00 | 07/07 - 13/08 | | 1 | | 0.11 |
|  | 12/05 - 16/07 | | 1 | 0.00 | 10/07 - 01/09 | | 3 | | 0.00 ± 0.00 |
|  | 20/05 - 11/07 | | 2 | 0.00 ± 0.00 | 07/07 - 11/09 | | 4 | | 0.03 ± 0.04 |
|  | 20/05 - 16/07 | | 7 | 0.066 ± 0.12 | 21/07 - 13/08 | | 2 | | 0.00 ± 0.00 |
|  | 29/05 - 11/07 | | 1 | 0.00 | 21/07 - 01/09 | | 6 | | 0.00 ± 0.00 |
|  | 29/05 - 16/07 | | 51 | 0.16 ± 0.31 | 21/07 - 11/09 | | 73 | | 0.03 ± 0.08 |
|  | 29/05 - 11/07 | | 3 | 0.00 ± 0.00 | 13/08 - 01/09 | | 5 | | 0.00 ± 0.00 |
|  | 11/06 - 16/07 | | 76 | 0.23 ± 0.47 | 13/08 - 11/09 | | 6 | | 0.00 ± 0.00 |
|  |  | |  |  | 02/09 - 11/09 | | 5 | | 0.00 ± 0.00 |
|  | **TOTAL**  ***C. segetum*** | | **144** | **0.18 ± 0.39** | **TOTAL**  ***C. jacea*** | | **105** | | **0.02 ± 0.07** |

|  | ***C. segetum*** | | | ***C. jacea*** | | |
| --- | --- | --- | --- | --- | --- | --- |
| **Predictor** | **Transect** | **Nb of individuals** | **Mean ± SD** | **Transect** | **Nb of individuals** | **Mean ± SD** |
| Foliar N content (%) | F1 | 9 | 1.53 ± 0.73 | F1 | 7 | 2.41 ± 1.11 |
|  | F10 | 9 | 3.32 ± 1.07 | F10 | 9 | 3.30 ± 0.41 |
|  | F2 | 9 | 1.63 ± 0.41 | F2 | 8 | 2.50 ± 0.45 |
|  | F3 | 9 | 2.97 ± 0.45 | F3 | 9 | 2.79 ±0.32 |
|  | F4 | 9 | 1.50 ± 0.28 | F4 | 8 | 2.52 ± 0.33 |
|  | F5 | 9 | 1.60 ± 0.30 | F5 | 9 | 2.09 ± 0.23 |
|  | F6 | 9 | 3.07 ± 0.77 | F6 | 6 | 1.67 ± 0.35 |
|  | F7 | 9 | 1.44 ± 0.18 | F7 | 1 | 2.12 |
|  | F8 | 9 | 3.47 ± 0.67 | F8 | 9 | 2.79 ± 0.28 |
|  | F9 | 9 | 3.24 ± 0.75 | F9 | 8 | 2.44 ± 0.38 |
|  | G1 | 9 | 2.55 ± 0.81 | G1 | 7 | 2.43 ± 0.34 |
|  | G2 | 9 | 2.88 ± 0.49 | G2 | 8 | 2.64 ± 0.59 |
|  | G3 | 9 | 2.64 ± 0.62 | G3 | 8 | 2.54 ± 0.55 |
|  | G4 | 9 | 1.36 ± 0.22 | G5 | 3 | 1.89 ± 0.31 |
|  | G5 | 9 | 1.62 ± 0.43 | G6 | 2 | 2.06 ± 0.57 |
|  | G6 | 9 | 1.54 ± 0.36 |  |  |  |
|  | **TOTAL**  ***C. segetum*** | **144** | **2.27 ± 0.96** | **TOTAL**  ***C. jacea*** | **102** | **2.51 ± 0.59** |
| Relative individual attractiveness (insect visits/individual/15 minutes) | F1 | 9 | 0.15 ± 0.12 | F1 | 8 | 0.04 ± 0.06 |
|  | F10 | 9 | 0.83 ± 0.57 | F10 | 9 | 0.25 ± 0.26 |
|  | F2 | 9 | 0.22 ± 0.15 | F2 | 8 | 0.21 ± 0.17 |
|  | F3 | 9 | 0.43 ± 0.29 | F3 | 9 | 0.37 ± 0.43 |
|  | F4 | 9 | 0.11 ± 0.11 | F4 | 8 | 0.04 ± 0.06 |
|  | F5 | 9 | 0.72 ± 0.39 | F5 | 9 | 0.27 ± 0.24 |
|  | F6 | 9 | 0.56 ± 0.35 | F6 | 6 | 0.33 ± 0.16 |
|  | F7 | 9 | 0.18 ± 0.09 | F7 | 1 | 0.00 |
|  | F8 | 9 | 2.20 ± 1.46 | F8 | 9 | 0.44 ± 0.19 |
|  | F9 | 9 | 1.05 ± 0.11 | F9 | 8 | 0.36 ± 0.22 |
|  | G1 | 9 | 0.10 ± 0.07 | G1 | 7 | 0.56 ± 0.47 |
|  | G2 | 9 | 0.41 ± 0.22 | G2 | 8 | 0.04 ± 0.05 |
|  | G3 | 9 | 0.77 ± 0.45 | G3 | 8 | 0.50 ± 0.59 |
|  | G4 | 9 | 0.25 ± 0.15 | G5 | 5 | 0.07 ± 0.07 |
|  | G5 | 9 | 0.67 ± 0.53 | G6 | 2 | 0.00 ± 0.00 |
|  | G6 | 9 | 0.74 ± 0.23 |  |  |  |
|  | **TOTAL**  ***C. segetum*** | **144** | **0.59 ± 0.67** | **TOTAL**  ***C. jacea*** | **105** | **0.27 ± 0.32** |

|  | ***C. segetum*** | | | ***C. jacea*** | | |
| --- | --- | --- | --- | --- | --- | --- |
| **Predictor** | **Transect** | **Nb of individuals** | **Mean ± SD** | **Transect** | **Nb of individuals** | **Mean ± SD** |
| Local species richness of potential pollinators | F1 | 9 | 7.11 ± 0.33 | F1 | 8 | 2.88 ± 1.55 |
|  | F10 | 9 | 15.78 ± 0.67 | F10 | 9 | 5.11 ± 2.26 |
|  | F2 | 9 | 6.67 ± 0.70 | F2 | 8 | 4.63 ± 1.06 |
|  | F3 | 9 | 11.11 ± 1.45 | F3 | 9 | 8.11 ± 2.67 |
|  | F4 | 9 | 8.11 ± 0.33 | F4 | 8 | 3.63 ± 1.06 |
|  | F5 | 9 | 10.33 ± 2.00 | F5 | 9 | 5.33 ± 1.32 |
|  | F6 | 9 | 10.88 ± 0.33 | F6 | 6 | 6.00 ± 2.45 |
|  | F7 | 9 | 3.56 ± 0.73 | F7 | 1 | 0.00 |
|  | F8 | 9 | 12.44 ± 2.60 | F8 | 9 | 8.00 ± 0.00 |
|  | F9 | 9 | 7.89 ± 1.76 | F9 | 8 | 10.00 ± 1.85 |
|  | G1 | 9 | 7.00 ± 0.00 | G1 | 7 | 10.43 ± 0.53 |
|  | G2 | 9 | 5.44 ± 0.53 | G2 | 8 | 0.75 ± 0.46 |
|  | G3 | 9 | 8.00 ± 0.00 | G3 | 8 | 9.00 ± 0.00 |
|  | G4 | 9 | 5.89 ± 1.27 | G5 | 5 | 5.00 ± 2.24 |
|  | G5 | 9 | 6.67 ± 1.00 | G6 | 2 | 2.00 ± 0.00 |
|  | G6 | 9 | 6.00 ± 0.00 |  |  |  |
|  | **TOTAL**  ***C. segetum*** | **144** | **8.31 ± 3.18** | **TOTAL**  ***C. jacea*** | **105** | **5.94 ± 3.22** |
| Local floral richness | F1 | 9 | 19.78 ± 6.96 | F1 | 8 | 12.75 ± 7.25 |
|  | F10 | 9 | 25.44 ± 1.67 | F10 | 9 | 14.56 ± 1.33 |
|  | F2 | 9 | 15.11 ± 2.42 | F2 | 8 | 8.38 ± 1.77 |
|  | F3 | 9 | 25.22 ± 2.64 | F3 | 9 | 14.11 ± 2.67 |
|  | F4 | 9 | 21.89 ±2.67 | F4 | 8 | 13.88 ± 3.18 |
|  | F5 | 9 | 18.33 ±0.50 | F5 | 9 | 14.22 ± 3.53 |
|  | F6 | 9 | 27.44 ± 1.67 | F6 | 6 | 12.67 ± 3.27 |
|  | F7 | 9 | 16.33 ± 1.58 | F7 | 1 | 0.00 |
|  | F8 | 9 | 28.67 ± 3.91 | F8 | 9 | 19.00 ± 0.00 |
|  | F9 | 9 | 24.56 ± 3.09 | F9 | 8 | 19.00 ± 0.00 |
|  | G1 | 9 | 10.67 ± 1.32 | G1 | 7 | 5.00 ± 0.00 |
|  | G2 | 9 | 13.89 ± 1.05 | G2 | 8 | 6.00 ± 1.85 |
|  | G3 | 9 | 16.67 ± 2.00 | G3 | 8 | 6.00 ± 0.00 |
|  | G4 | 9 | 19.33 ± 1.58 | G5 | 5 | 5.4 ± 1.34 |
|  | G5 | 9 | 18.44 ± 3.09 | G6 | 2 | 13.00 ± 0.00 |
|  | G6 | 9 | 22.33 ± 2.00 |  |  |  |
|  | **TOTAL**  ***C. segetum*** | **144** | **20.26 ± 5.64** | **TOTAL**  ***C. jacea*** | **105** | **11.90 ± 5.47** |

|  | ***C. segetum*** | | | ***C. jacea*** | | |
| --- | --- | --- | --- | --- | --- | --- |
| **Predictor** | **Transect** | **Nb of individuals** | **Mean ± SD** | **Transect** | **Nb of individuals** | **Mean ± SD** |
| Local floral density (/m²) | F1 | 9 | 77.75 ± 6.23 | F1 | 8 | 60,36 ± 0,53 |
|  | F10 | 9 | 83.21 ± 2.87 | F10 | 9 | 144,5 ± 18,67 |
|  | F2 | 9 | 54.28 ± 23.82 | F2 | 8 | 11,56 ± 3,86 |
|  | F3 | 9 | 475.22 ± 74.49 | F3 | 9 | 284,22 ± 57,08 |
|  | F4 | 9 | 76.03 ± 3.42 | F4 | 8 | 50,81 ± 0,08 |
|  | F5 | 9 | 137.16 ± 4.16 | F5 | 9 | 134,70 ± 2,90 |
|  | F6 | 9 | 237.01 ± 2.43 | F6 | 6 | 268,26 ± 0,53 |
|  | F7 | 9 | 70,81 ± 3,79 | F7 | 1 | 0.00 |
|  | F8 | 9 | 993,92 ± 154,62 | F8 | 9 | 279.00 ± 0.00 |
|  | F9 | 9 | 539,26 ± 29,33 | F9 | 8 | 245,42 ± 52,59 |
|  | G1 | 9 | 80,55 ± 6,77 | G1 | 7 | 38,35 ± 5,57 |
|  | G2 | 9 | 593,15 ± 49,08 | G2 | 8 | 295,26 ± 114,47 |
|  | G3 | 9 | 360,43 ± 3,80 | G3 | 8 | 152,54 ± 0.00 |
|  | G4 | 9 | 48,93 ± 5,36 | G5 | 5 | 0,60 ± 0,33 |
|  | G5 | 9 | 50,08 ± 14,61 | G6 | 2 | 51,53 ± 0.00 |
|  | G6 | 9 | 195,81 ± 9,21 |  |  |  |
|  | **TOTAL**  ***C. segetum*** | **144** | **2,27 ± 0,96** | **TOTAL**  ***C. jacea*** | **105** | **2,51 ± 0,59** |
| Local density of potential pollen donors (/m²) | F1 | 9 | 0.00 ± 0.00 | F1 | 8 | 0.00 ± 0.00 |
|  | F10 | 9 | 0,21 ± 0,02 | F10 | 9 | 0,04 ± 0,03 |
|  | F2 | 9 | 0.00 ± 0.00 | F2 | 8 | 0.00 ± 0.00 |
|  | F3 | 9 | 0.00 ± 0.00 | F3 | 9 | 0.00 ± 0.00 |
|  | F4 | 9 | 0.00 ± 0.00 | F4 | 8 | 0.00 ± 0.00 |
|  | F5 | 9 | 0,71 ± 0,01 | F5 | 9 | 0.00 ± 0.00 |
|  | F6 | 9 | 0.00 ± 0.00 | F6 | 6 | 0.00 ± 0.00 |
|  | F7 | 9 | 0,32 ± 0,02 | F7 | 1 | 0.00 |
|  | F8 | 9 | 0,11 ± 0,05 | F8 | 9 | 0,24 ± 0.00 |
|  | F9 | 9 | 1,51 ± 0,08 | F9 | 8 | 0.00 ± 0.00 |
|  | G1 | 9 | 0.00 ± 0.00 | G1 | 7 | 0.00 ± 0.00 |
|  | G2 | 9 | 0.00 ± 0.00 | G2 | 8 | 0.00 ± 0.00 |
|  | G3 | 9 | 0.00 ± 0.00 | G3 | 8 | 0.00 ± 0.00 |
|  | G4 | 9 | 0.00 ± 0.00 | G5 | 5 | 0.00 ± 0.00 |
|  | G5 | 9 | 0.00 ± 0.00 | G6 | 2 | 0.00 ± 0.00 |
|  | G6 | 9 | 0.00 ± 0.00 |  |  |  |
|  | **TOTAL**  ***C. segetum*** | **144** | **0,18 ± 0,39** | **TOTAL**  ***C. jacea*** | **105** | **0,02 ± 0,07** |

**
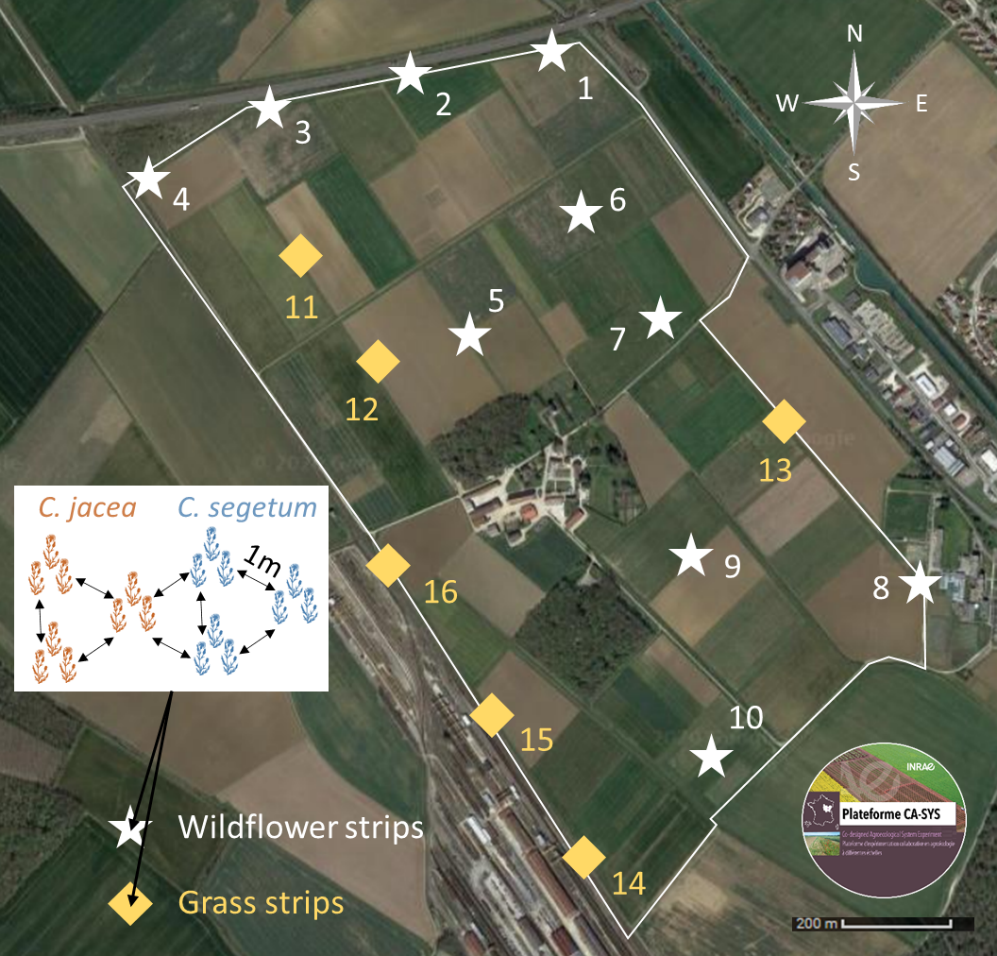
**

**Figure S1**. Location of the 16 focal plots established on the CA-SYS agroecological platform of the INRAE U2E experimental unit. Each plot was constituted of three triplets of focal *C. segetum* individuals and three triplets of focal *C. jacea* individuals. Ten plots were established in sown wildflower field borders (1-10, white stars) and six in sown grassy field borders (11-16, yellow diamonds). All plots were located at least 150m-apart from each other.

**Equations S1**

Mean precipitation (mm)


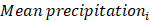


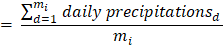


Mean temperature (°C)


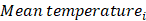


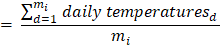


Where:


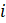
 = the focal individual considered


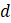
 = a single day comprised between the beginning of the flowering period of individual
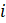
 and harvest


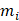
 = the total duration (days) of the reproduction period of individual
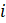
 (flowering period + period of seed maturation before harvest)

**Equation S2**

Mean local floral richness


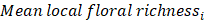


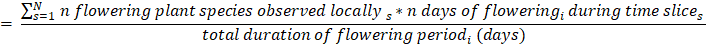


Where:


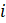
 = the focal individual considered


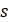
 = a single floral survey conducted in the local plant community (transect) surrounding
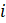
 during the flowering period of individual
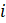


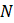
 = the total number of floral surveys conducted during the flowering period of individual
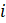


To calculate the mean local floral richness experienced by the individual
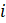
 during its flowering period, we multiplied, for each floral survey
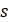
 conducted during the flowering period of individual
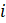
, the total number of flowering plant species observed in bloom during the survey
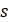
 on the transect surrounding
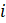
, and the number of days of the flowering period of individual
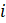
 that were covered by the time slice during which survey
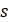
 was considered as representative. We then summed up the results obtained across all floral surveys conducted during the flowering period of individual
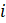
, and divided the total by the duration (in days) of the flowering period of individual
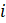
.

**Equation S3**

Mean local floral density (/m²)


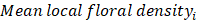


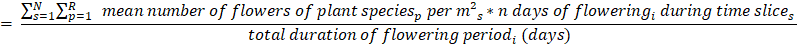


Where:


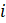
 = the focal individual considered


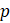
 = a single flowering plant species present in the local floral community of individual
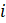


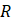
 = the total number of flowering plant species present in the local floral community of individual
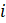


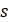
 = a single floral survey conducted in the local plant community (transect) surrounding
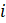
 during the flowering period of individual
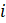


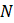
 = the total number of floral surveys conducted during the flowering period of individual
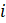


**References S1**

Audibert C. (2001). Notes sur les Vespidae : biologie, systématique et clé d’identification des espèces et la biologie et la détermination des Polistes. Bull. 16 et 17 du Club Rosalia.

Amiet F., Herrmann M., Müller A. & Neumeyer R. (2001). Fauna Helvetica 6 : Apidae 3 : Halictus, Lasioglossum.  Centre Suisse de Cartographie de la Faune & Schweizerische Entomologische Gesellschaft, Neuchâtel, 208 p.

Amiet F., Herrmann M., Müller A. & Neumeyer R. (2004). Fauna Helvetica 9 : Apidae 4 : Anthidium, Chelostoma, Coelioxys, Dioxys, Heriades, Lithurgus, Megachile, Osmia, Stelis.  Centre Suisse de Cartographie de la Faune & Schweizerische Entomologische Gesellschaft, Neuchâtel, 273 p.

Patiny S. & Terzo M. (2010). Catalogue et clé des sous-genres et espèces du genre *Andrena* de Belgique et du Nord de la France (Hymenoptera, Apoidea). 39 p.

Pauly A. (2015). Clés illustrées pour l’identification des abeilles de Belgique et des régions limitrophes (Hymenoptera: Apoidea). I. Halictidae. Document de Travail du Projet BELBEES, 14 novembre 2015, 118 p.

Pauly A. (2015). Clés illustrées pour l’identification des abeilles de Belgique et des régions limitrophes (Hymenoptera: Apoidea). II. Megachilidae. Document de Travail du Projet BELBEES, 7 septembre 2015, 61 p.

Pauly A. (2016). Les espèces du genre *Sphecodes* Latreille, 1804, en Belgique (Hymenoptera, Apoidea, Halictidae). Document de Travail du Projet BELBEES, 22 septembre 2016, 93 p.

Rasmont P. & Terzo M. (2017). Catalogue et clé des sous-genre et espèces du genre Bombus de Belgique et du nord de la France (Hymenoptera, Apoidea), 2ème édition, 26 p.

Speight M.C.D., Withers P. & Dussaix C. (2015). Clé StN pour la détermination des genres de Syrphidae européens 2015. Syrph the Net, the database of European Syrphidae (Diptera) 81. Syrph the Net publications, Dublin, Ireland.

Speight M.C.D. & Sarthou J.-P. (2013). Clé StN pour la détermination des adultes de Syrphidae européens 2013. Syrph the Net, the database of European Syrphidae (Diptera) 74. Syrph the Net publications, Dublin, Ireland.

Stubbs A.E. & Falks S. (2002). British Hoverflies: an illustrated identification guide. 2^nd^. British Entomological and Natural History Society. 469 pp.
